# Supplementary material for: Is routine Vitamin A supplementation still justified for children in Nepal? Trial synthesis findings applied to Nepal national mortality estimates
Source: PLoS One. 2022 May 18;17(5):e0268507. doi: 10.1371/journal.pone.0268507 (PMC9116662; doi:10.1371/journal.pone.0268507)
Supplement: S1 Checklist — (DOCX) [file pone.0268507.s001.docx]

| **Section and Topic** | **Item #** | **Checklist item** | **Location where item is reported** |
| --- | --- | --- | --- |
| **TITLE** | | |  |
| Title | 1 | Identify the report as a systematic review. | Pg.1 |
| **ABSTRACT** | | |  |
| Abstract | 2 | See the PRISMA 2020 for Abstracts checklist. | Pg. 2-3 |
| **INTRODUCTION** | | |  |
| Rationale | 3 | Describe the rationale for the review in the context of existing knowledge. | Pg. 4-5 |
| Objectives | 4 | Provide an explicit statement of the objective(s) or question(s) the review addresses. | Pg. 5 |
| **METHODS** | | |  |
| Eligibility criteria | 5 | Specify the inclusion and exclusion criteria for the review and how studies were grouped for the syntheses. | Pg. 5-6 (Details in  S1 Appendix) |
| Information sources | 6 | Specify all databases, registers, websites, organisations, reference lists and other sources searched or consulted to identify studies. Specify the date when each source was last searched or consulted. | Pg. 6  (Details in  S1 Appendix) |
| Search strategy | 7 | Present the full search strategies for all databases, registers and websites, including any filters and limits used. | Pg. 6  (Details in  S1 Appendix) |
| Selection process | 8 | Specify the methods used to decide whether a study met the inclusion criteria of the review, including how many reviewers screened each record and each report retrieved, whether they worked independently, and if applicable, details of automation tools used in the process. | S1 Appendix |
| Data collection process | 9 | Specify the methods used to collect data from reports, including how many reviewers collected data from each report, whether they worked independently, any processes for obtaining or confirming data from study investigators, and if applicable, details of automation tools used in the process. | Pg. 6  (Details in  S1 Appendix) |
| Data items | 10a | List and define all outcomes for which data were sought. Specify whether all results that were compatible with each outcome domain in each study were sought (e.g. for all measures, time points, analyses), and if not, the methods used to decide which results to collect. | S1 Appendix |
|  | 10b | List and define all other variables for which data were sought (e.g. participant and intervention characteristics, funding sources). Describe any assumptions made about any missing or unclear information. | S1 Appendix |
| Study risk of bias assessment | 11 | Specify the methods used to assess risk of bias in the included studies, including details of the tool(s) used, how many reviewers assessed each study and whether they worked independently, and if applicable, details of automation tools used in the process. | Pg. 6  (Details in  S1 Appendix) |
| Effect measures | 12 | Specify for each outcome the effect measure(s) (e.g. risk ratio, mean difference) used in the synthesis or presentation of results. | S1 Appendix |
| Synthesis methods | 13a | Describe the processes used to decide which studies were eligible for each synthesis (e.g. tabulating the study intervention characteristics and comparing against the planned groups for each synthesis (item #5)). | S1 Appendix |
|  | 13b | Describe any methods required to prepare the data for presentation or synthesis, such as handling of missing summary statistics, or data conversions. | S1 Appendix |
|  | 13c | Describe any methods used to tabulate or visually display results of individual studies and syntheses. | S1 Appendix |
|  | 13d | Describe any methods used to synthesize results and provide a rationale for the choice(s). If meta-analysis was performed, describe the model(s), method(s) to identify the presence and extent of statistical heterogeneity, and software package(s) used. | Pg. 7  (Details in  S1 Appendix) |
|  | 13e | Describe any methods used to explore possible causes of heterogeneity among study results (e.g. subgroup analysis, meta-regression). | Pg. 7  (Details in  S1 Appendix |
|  | 13f | Describe any sensitivity analyses conducted to assess robustness of the synthesized results. | S1 Appendix |
| Reporting bias assessment | 14 | Describe any methods used to assess risk of bias due to missing results in a synthesis (arising from reporting biases). | S1 Appendix |
| Certainty assessment | 15 | Describe any methods used to assess certainty (or confidence) in the body of evidence for an outcome. | Pg. 7  (Details in  S1 Appendix) |
| **RESULTS** | | |  |
| Study selection | 16a | Describe the results of the search and selection process, from the number of records identified in the search to the number of studies included in the review, ideally using a flow diagram. | Pg. 7  Results |
|  | 16b | Cite studies that might appear to meet the inclusion criteria, but which were excluded, and explain why they were excluded. | S2 Appendix |
| Study characteristics | 17 | Cite each included study and present its characteristics. | Pg. 8-10  Included studies  (Details in  S3 Appendix) |
| Risk of bias in studies | 18 | Present assessments of risk of bias for each included study. | Pg. 10-11  Risk of bias  (Details in  S3 Appendix) |
| Results of individual studies | 19 | For all outcomes, present, for each study: (a) summary statistics for each group (where appropriate) and (b) an effect estimate and its precision (e.g. confidence/credible interval), ideally using structured tables or plots. | Pg. 11  Effect of intervention |
| Results of syntheses | 20a | For each synthesis, briefly summarise the characteristics and risk of bias among contributing studies. | Pg. 12  Sensitivity analyses |
|  | 20b | Present results of all statistical syntheses conducted. If meta-analysis was done, present for each the summary estimate and its precision (e.g. confidence/credible interval) and measures of statistical heterogeneity. If comparing groups, describe the direction of the effect. | Pg. 11-14 |
|  | 20c | Present results of all investigations of possible causes of heterogeneity among study results. | Pg. 11-14 |
|  | 20d | Present results of all sensitivity analyses conducted to assess the robustness of the synthesized results. | Pg. 12  Sensitivity analyses |
| Reporting biases | 21 | Present assessments of risk of bias due to missing results (arising from reporting biases) for each synthesis assessed. | Pg. 12 |
| Certainty of evidence | 22 | Present assessments of certainty (or confidence) in the body of evidence for each outcome assessed. | Pg. 15-16  Table 3  S1 Table |
| **DISCUSSION** | | |  |
| Discussion | 23a | Provide a general interpretation of the results in the context of other evidence. | Pg. 17  Summary of main results |
|  | 23b | Discuss any limitations of the evidence included in the review. | We have analysed the applicability about the context of Nepal |
|  | 23c | Discuss any limitations of the review processes used. | None |
|  | 23d | Discuss implications of the results for practice, policy, and future research. | Pg. 17-18 |
| **OTHER INFORMATION** | | |  |
| Registration and protocol | 24a | Provide registration information for the review, including register name and registration number, or state that the review was not registered. | Pg. 6  Methods  Data collection and analysis |
|  | 24b | Indicate where the review protocol can be accessed, or state that a protocol was not prepared. | Pg. 6 |
|  | 24c | Describe and explain any amendments to information provided at registration or in the protocol. | None |
| Support | 25 | Describe sources of financial or non-financial support for the review, and the role of the funders or sponsors in the review. | This work is funded by the Research Evidence and Development Initiative (READ-It), which is an accountable grant of UK aid through the Foreign, Commonwealth and Development Office (FCDO) (Project number 300342-104). The funder has no role in design and execution of review. |
| Competing interests | 26 | Declare any competing interests of review authors. | The authors declare that they have no competing interest |
| Availability of data, code and other materials | 27 | Report which of the following are publicly available and where they can be found: template data collection forms; data extracted from included studies; data used for all analyses; analytic code; any other materials used in the review. | All supporting documents are provided as supporting information and appendixes |

*From:*  Page MJ, McKenzie JE, Bossuyt PM, Boutron I, Hoffmann TC, Mulrow CD, et al. The PRISMA 2020 statement: an updated guideline for reporting systematic reviews. BMJ 2021;372:n71. doi: 10.1136/bmj.n71

For more information, visit: <http://www.prisma-statement.org/>
