# Supplementary figures and images for: Is routine Vitamin A supplementation still justified for children in Nepal? Trial synthesis findings applied to Nepal national mortality estimates
Source: PLoS One. 2022 May 18;17(5):e0268507. doi: 10.1371/journal.pone.0268507 (PMC9116662; doi:10.1371/journal.pone.0268507)

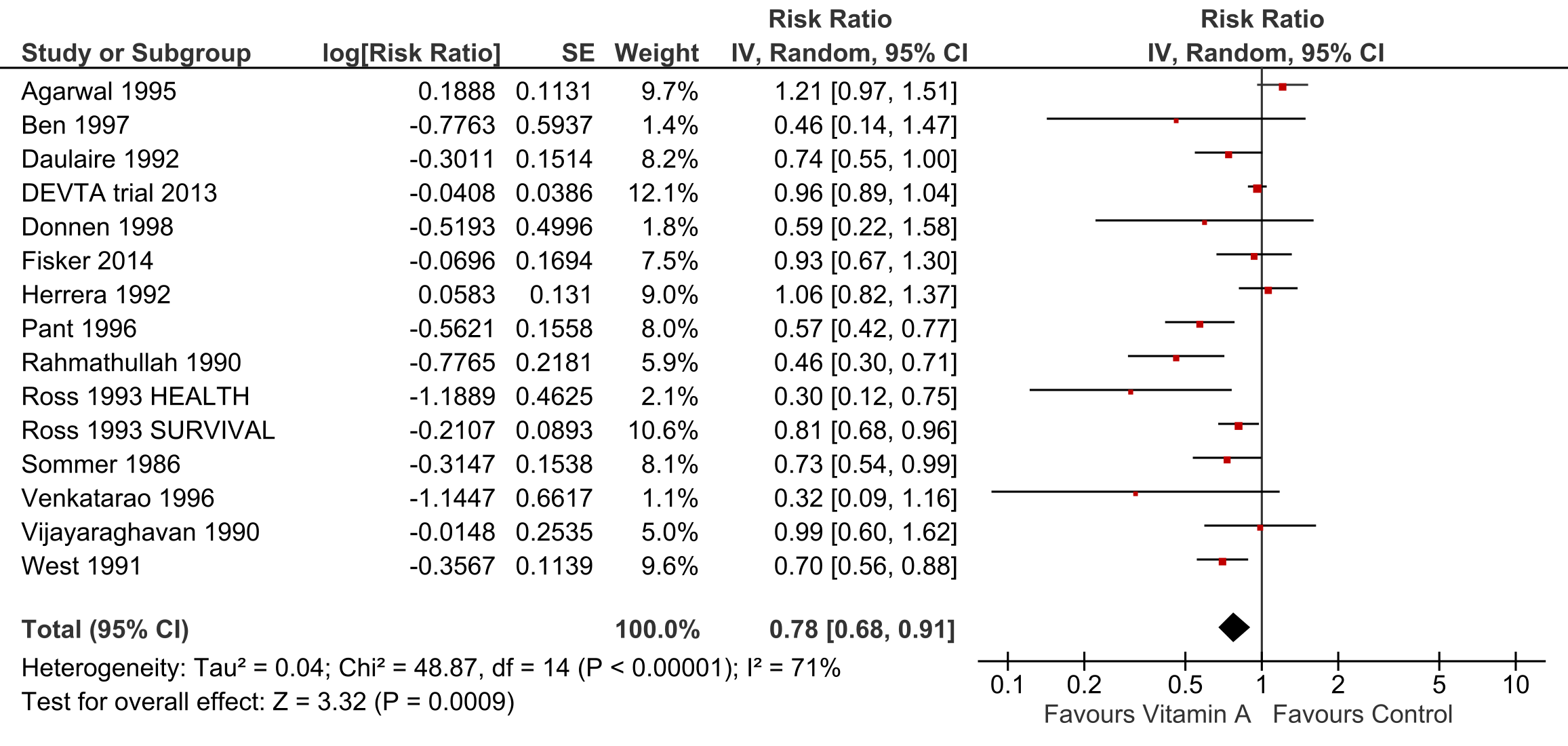

Supplement: S1 Fig — (TIFF) [file pone.0268507.s002.tiff]

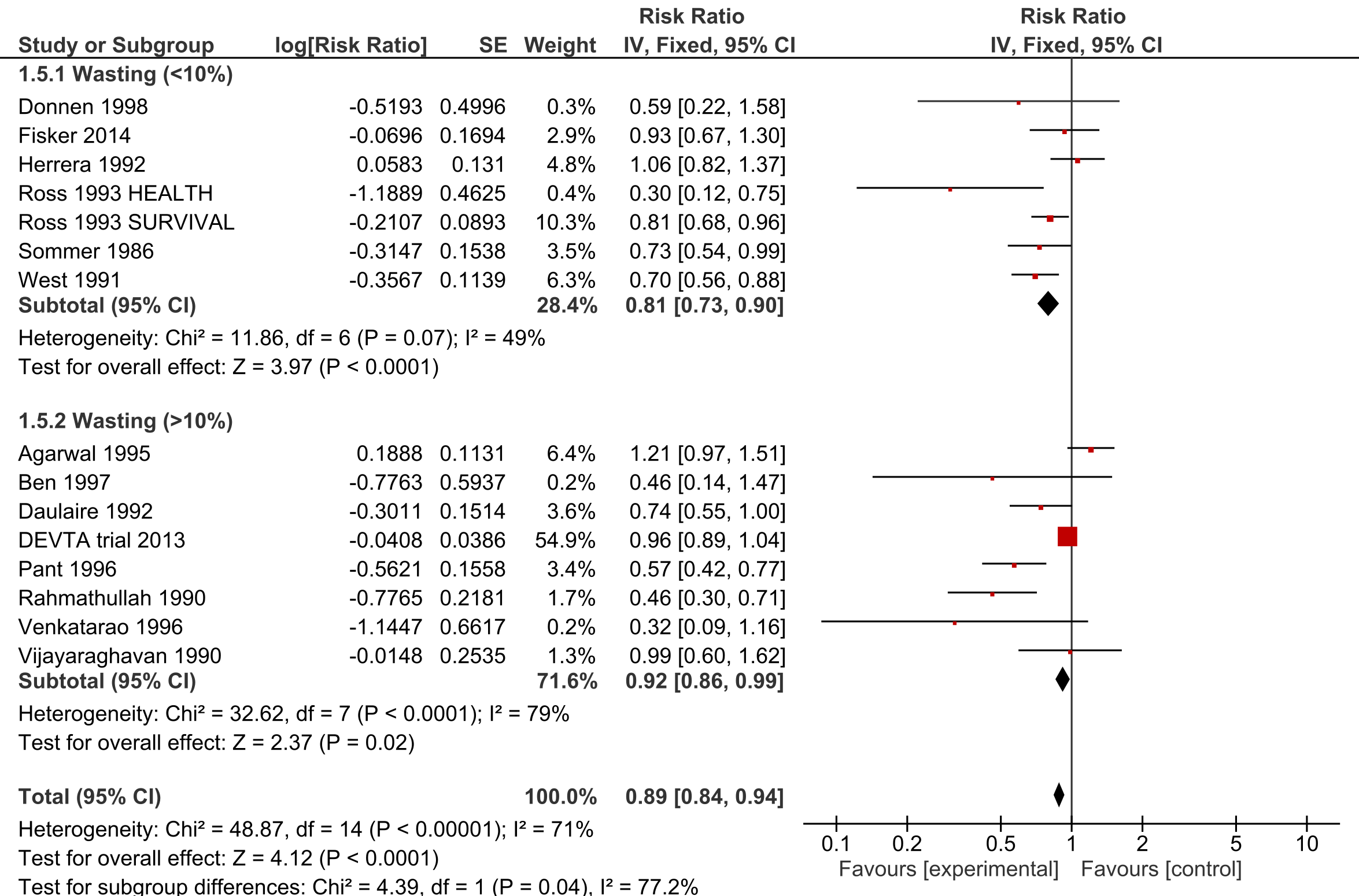

Supplement: S2 Fig — (TIFF) [file pone.0268507.s003.tiff]

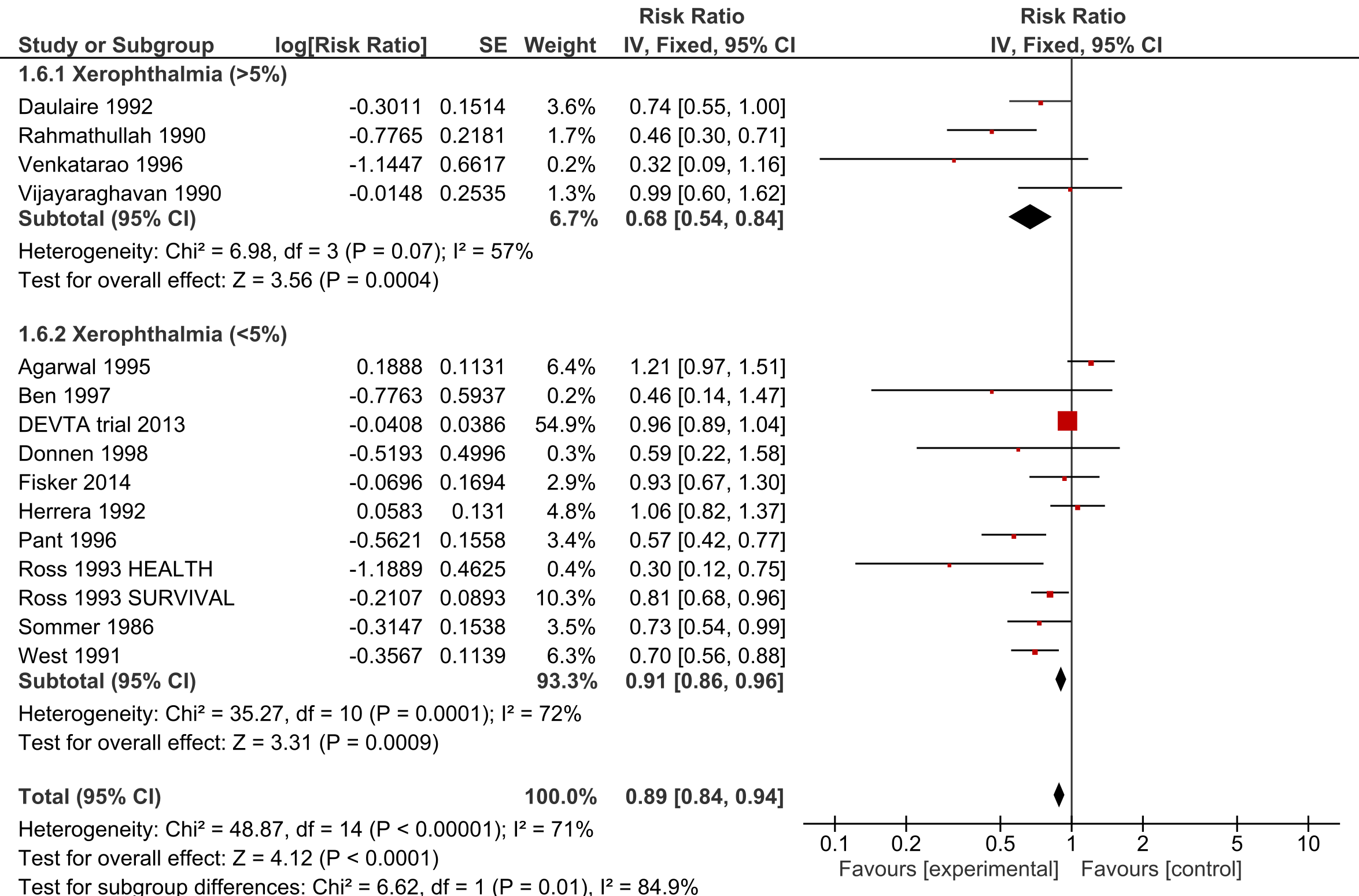

Supplement: S3 Fig — (TIFF) [file pone.0268507.s004.tiff]

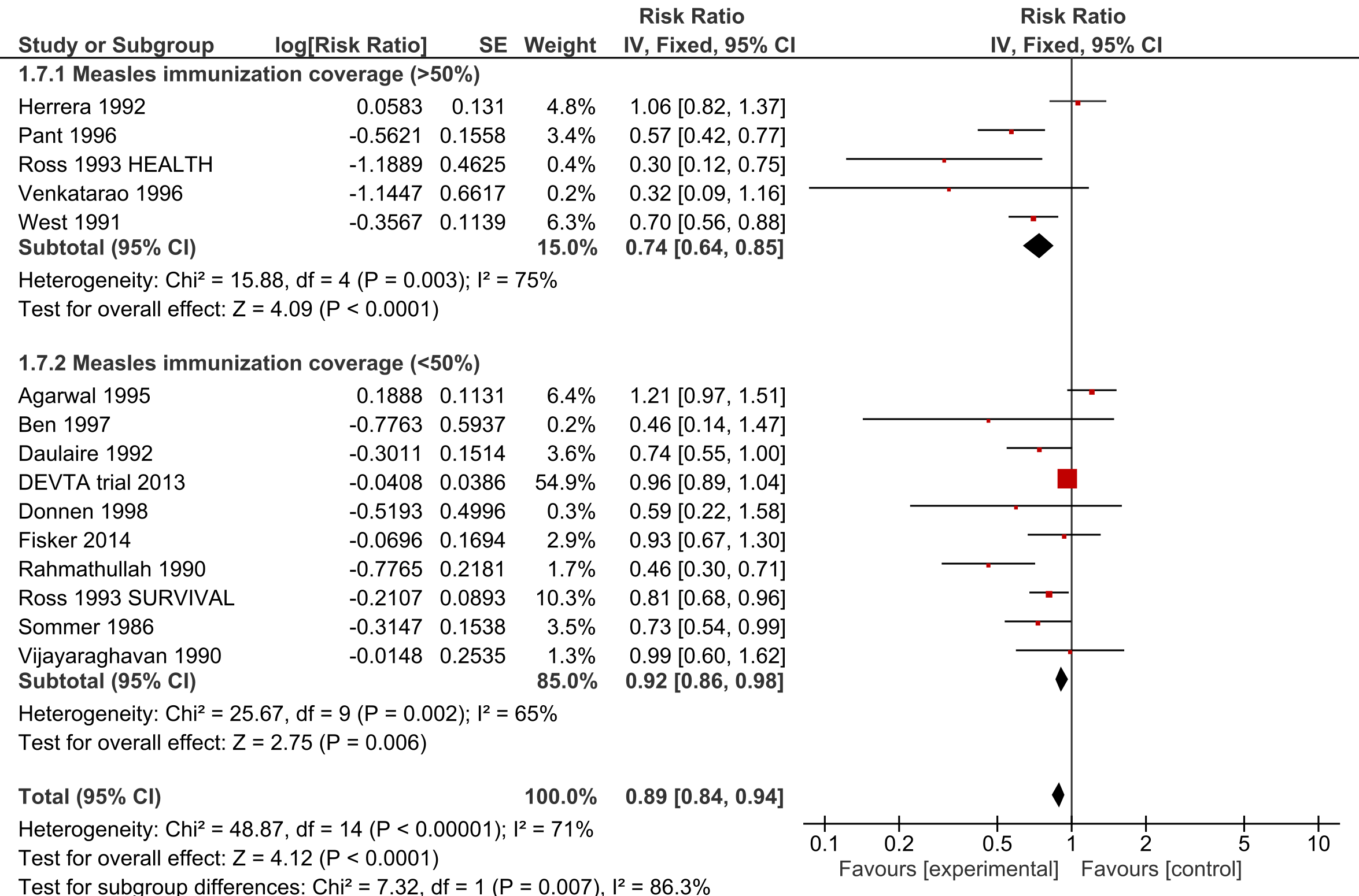

Supplement: S4 Fig — (TIFF) [file pone.0268507.s005.tiff]

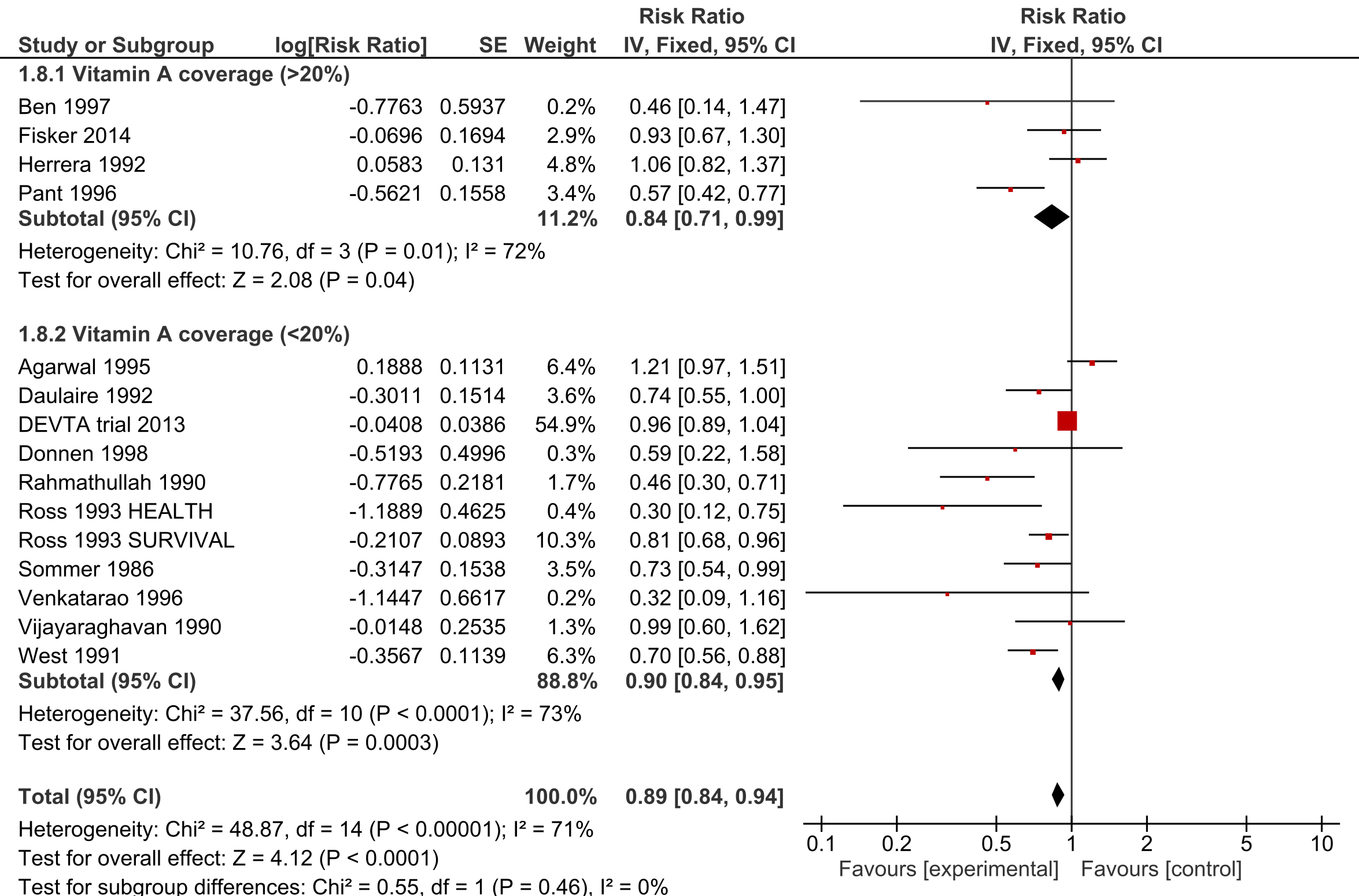

Supplement: S5 Fig — (TIFF) [file pone.0268507.s006.tiff]
