# Supplementary material for: Is routine Vitamin A supplementation still justified for children in Nepal? Trial synthesis findings applied to Nepal national mortality estimates
Source: PLoS One. 2022 May 18;17(5):e0268507. doi: 10.1371/journal.pone.0268507 (PMC9116662; doi:10.1371/journal.pone.0268507)
Supplement: S1 Table — (DOCX) [file pone.0268507.s007.docx]

Table 1: **Summary of findings (GRADE methods) estimating effects of blanket Vitamin A supplementation in Nepal at the sub-national level. Applying the best estimate of effect to sub-national statistics of Nepal for Under-Five Mortality Rate**

| **Outcomes** | | **Anticipated absolute effects* (95% CI)** | | **Relative effect (95% CI)** | **Absolute (95% CI)** | **№ of participants (studies)** | **Certainty of the evidence (GRADE)** | **Comments** |
| --- | --- | --- | --- | --- | --- | --- | --- | --- |
|  |  | **Risk with control** | **Risk with Vitamin A supplementation** |  |  |  |  |  |
| **All-cause child mortality** | | **19 per 1,000**  U5MR in the least deprived province in Nepal | **17 per 1,000** (16 to 18) | **RR 0.91** (0.85 to 0.97) | **2 fewer per 1,000** (from 3 fewer to 1 fewer) | 1,046,829 (6 RCTs) | ⨁⨁◯◯ Low^a,b^ | Vitamin A supplementation may result in a small reduction in child mortality |
|  |  | **40 per 1,000**  U5MR in the deprived province in Nepal | **36 per 1,000** (34 to 39) |  | **4 fewer per 1,000** (from 6 fewer to 1 fewer) |  |  |  |
|  | * **The risk in the intervention group** (and its 95% confidence interval) is based on the assumed risk in the comparison group and the **relative effect** of the intervention (and its 95% CI).  **CI:** confidence interval; **RR:** risk ratio **U5MR:** Under Five Mortality Rate | | | | | | | |
|  | **GRADE Working Group grades of evidence** **High certainty:** we are very confident that the true effect lies close to that of the estimate of the effect. **Moderate certainty:** we are moderately confident in the effect estimate: the true effect is likely to be close to the estimate of the effect, but there is a possibility that it is substantially different. **Low certainty:** our confidence in the effect estimate is limited: the true effect may be substantially different from the estimate of the effect. **Very low certainty:** we have very little confidence in the effect estimate: the true effect is likely to be substantially different from the estimate of effect. | | | | | | | |

#### **Explanations**

a. Downgraded 1 level due to serious imprecision (Effect estimate includes both negligible effect (3% reduction) and considerable benefit (15% reduction) with vitamin A supplementation).

b. Downgraded 1 level due to serious inconsistency (I^2^ was 75%, and the results of Rahmathullah 1990; Ross 1993 HEALTH and Ross 1993 SURVIVAL demonstrated evidence of benefit contrary to the results of other studies)
