# Supplementary material for: Is routine Vitamin A supplementation still justified for children in Nepal? Trial synthesis findings applied to Nepal national mortality estimates
Source: PLoS One. 2022 May 18;17(5):e0268507. doi: 10.1371/journal.pone.0268507 (PMC9116662; doi:10.1371/journal.pone.0268507)
Supplement: S1 Appendix — (DOCX) [file pone.0268507.s008.docx]

# S1: METHODS

## Criteria for considering studies for this review

### Types of studies

We considered randomized controlled trials (RCTs) and cluster RCTs for assessing the effects of VAS in reducing child mortality.

### Types of participants

We included children who were aged six months to five years at the time of recruitment. We excluded children with any infection or disease and children in hospitals.

### Types of interventions

*Intervention*

For intervention, we considered oral vitamin A supplements with various doses and frequencies. We initially considered including VAS doses similar to WHO recommendation (dose of 100000 IU Vitamin A in infants 6–11 months of age (once) and 200000 IU in children 12–59 months of age). Since many studies were conducted before there was a WHO recommendation about VAS and since we mainly intended to evaluate the effect of VAS on mortality, we included studies with variable doses and frequencies.

*Control*

Placebo or no intervention.

We considered co-interventions studies only if the interventions are identical in both groups. When a trial included more than one intervention group, we combined the groups for the main analysis. Likewise, if a trial had more than one control group, we only considered one relevant group as the control group.

### Types of outcome measures

We extracted the following outcome measures from the included studies.

*Primary outcome*

- All-cause mortality in children six months to five years of age.

## Search methods for identification of studies

### Electronic searches

We used the same search strategies as used in the review by Imdad et al., 2017 to identify any new relevant studies (Imdad et al., 2017). Accordingly, using the same strategies we searched across different databases and trials registers such as Cochrane Central Register of Controlled Trials, MEDLINE, EMBASE, meta register of Clinical Trials, Web of Science, Global Health, Latin American Database (LILACS), and African Index Medicus.

### Searching other resources

We also checked the reference lists of relevant studies to find additional potential studies, not listed by previous searches. We also explored the World Health Organization International Clinical Trials Registry (ICTRP) and ClinicalTrials.gov to identify any other ongoing trials.

##

## Data collection and analysis

### Selection of studies

Two review authors (Khem Narayan Pokhrel and Samjhana Shrestha) independently assessed the studies and screened titles and abstracts for inclusion in the review. We used covidence, web software (Covidence, 2021) to organize, and manage search results and used it for removing duplicates and screening titles and abstracts. For studies whose relevance cannot be established through titles or abstracts screening, we retrieved and reviewed full texts of these studies. For studies reporting multiple time points, we extracted information from the longest follow-up period for the outcome of mortality. We resolved the difference in opinions regarding study selection for inclusion by discussion. In case of indecision, we consulted a third reviewer. Excluded studies were listed indicating the reasons for exclusion.

###

### Data extraction and management

Two reviewer authors (Khem Narayan Pokhrel and Samjhana Shrestha) used a published data extraction form (CRD, 2009) to extract the data separately using the agreed form. Consensus with the extracted data was maintained through discussion and discrepancies were resolved through consultation with a third review author. We extracted the following information from each of the included studies using the data extraction form:

- Study details: Design, setting, study year, year of publication, method of recruitment, inclusion and exclusion criteria, unit of analysis, allocation ratio, risk of bias
- Participant details: socio-demographic characteristics, the sample size
- Intervention and comparison details: dose, duration, frequency route, co-intervention if any, placebo/control details.
- Outcomes details: outcome definition, loss to follow up, adverse events
- Miscellaneous: conclusions, relevant references

**Extracting data on potential effect modifiers**

We also extracted data on the potential secular effect modifiers which may influence mortality estimates of the trials. Examples of possible effect modifiers have been described above taking Nepal as an example. We described the trials with reference to these effect modifiers. If data on such effect modifiers were not reported in the trials, we looked for estimates of such modifiers in different sources such as the Global Health Observatory data repository (WHO, 2021) and other potential data sources (World Bank, 2021). We considered the following factors as potential effect modifiers and extracted

***Child mortality and morbidity rates:***

- Under Five Mortality Rate (U5MR)
- Infant Mortality Rate (IMR)
- Measles Mortality Rate
- Diarrhoea Mortality Rate
- Incidence of measles
- Incidence of diarrhoea

***Child nutrition status***

- Prevalence of stunting, wasting and underweight
- Vitamin A deficiency: Vitamin A serum retinol, xerophthalmia: Night-blindness

***Health service delivery***

- Measles immunization coverage
- Vitamin A supplementation coverage

### Assessment of risk of bias in included studies

Two reviewer authors (Khem Narayan Pokhrel and Samjhana Shrestha) independently assessed the methodological quality of the included studies based on the criteria detailed in the Cochrane Risk of Bias Tool (Higgins et al., 2011). Using this tool, we assessed each study based on criteria such as the method of sequence generation; allocation concealment; blinding of participants, providers, and outcomes assessors; addressing incomplete outcomes, and selective outcome reporting. For cluster RCTs, we additionally assessed other criteria such as the possibility of bias arising due to recruitment of participants into clusters; baseline imbalance; loss of clusters; incorrect analysis; and comparability with individually randomized trials. We graded each study as having a high, low or unclear risk of bias thereby providing supporting judgements for each criterion assessed.

### Measures of treatment effect

We considered adjusted estimates from the cluster-RCTs where trial authors reported such estimates. When such estimates are not reported, we extracted raw values (number of events, number of children randomized or number of child-years). For individual RCTs, we mostly used raw mortality data reported in the trials.

### Unit of analysis issues

In cluster RCTS where units of randomization are clusters rather than individuals, we followed statistical methods recommended for analyzing clustered data in the Cochrane Handbook for Systematic Reviews of Interventions (Higgins et al., 2021). For studies that did report cluster adjustments process or intra-cluster correlation coefficient (ICC), we attempted to contact the trial authors. However, we were not able to get responses from the trial authors. So we used the design effects used in the previous review (Beaton et al., 1993) to adjust for clustering in those trials which did not control for clustering.

**Multiple-arm trials**

In the case of multiple-arm trials, we grouped data making sure that Vitamin A supplementation is the only difference between the groups being compared. For instance, if a trial has four arms (Vitamin A alone, Albendazole, Vitamin A plus Albendazole and placebo), we considered it vitamin A alone versus placebo. When the trials used different doses in multiple-arm trials, we combined the groups with different doses to avoid the double-counting of the children in the control group.

### Dealing with missing data

We contacted trial authors to get information relating to missing data concerning the trials. However, the trial authors were unable to provide information on such data. So, we described such trials by detailing dropout across the study groups. For studies reporting incomplete data, we conducted a sensitivity analysis to assess if these studies have any impact on the results of the meta-analysis.

### Assessment of heterogeneity

Two review authors assessed clinical heterogeneity of the included studies by examining the distribution of factors based on participants, intervention (dose, duration and co-interventions), study setting, and outcome. Likewise, we assessed methodological heterogeneity by assessing the risk of bias of each included study (See ‘Risk of bias assessment in Characteristics of Included studies table). Similarly, we conducted a visual inspection of the forest plots, observed the Chi2 test and associated p-value, and the I2 statistics of the analyses to evaluate the presence of statistical heterogeneity. We considered the heterogeneity to be substantial when the P-value <0.10 and I2 > 50% and visual inspection of forest plots indicated the possible presence of heterogeneity. We also reported Tau2 – an estimate of between-study variance.

### Assessment of reporting biases

For the outcome of mortality which consisted of more than 10 studies, we generated a funnel plot to assess the possibility of publication bias through visual inspection of the funnel plot for asymmetry. For ease of understanding, we plotted the funnel plot including three categories such as trials with less than 1000 participants, trials with 1000 to up to 2000 participants and trials with greater than 2000 participants.

## Data synthesis

We conducted a meta-analysis following the *Cochrane Handbook for Systematic Reviews of Interventions* (Deeks et al., 2020) using RevMan version 5.4 software (Review Manager, 2020). Data extracted from the trials were in various formats. So, we used the generic variance (GIV) option in Revman. With the GIV option, we were able to directly enter the summary estimates such as risk ratio or rate ratio from the trials which did not report numbers for numerators and denominators required for calculating the summary estimate. Also, GIV allowed the calculation of log of effect size (RR) and standard error (SE), so we used the built-in calculator in RevMan to enter the data and calculate the log of RR and SE. We used a fixed-effect model and weighted overall effects by the inverse of the variance and reported the effect estimate with 95% confidence intervals.

### Subgroup analysis and investigation of heterogeneity

Several factors -individual-level factors (age, gender etc.) and trial-level factors (background parameters of the trials) influences the effectiveness of an intervention. We assessed the effects of such factors, particularly the trial level factors which we have identified as potential effect modifiers possibly modifying the effect of VAS on mortality. As pre-specified, we performed subgroup analyses when data on potential effect modifiers were available for the trials. Not all pre-specified subgroup analyses were conducted given the lack of data on effect modifiers across the trials. Accordingly, the following subgroup analyses were performed:

**Child mortality:**

- Subgroup analysis by decade: We included this subgroup to assess the effect of VAS over the periods.
- Subgroup analysis according to background U5MR: We performed subgroup analysis by background U5MR and used the cut-off criteria that reflected both the current U5MR in Nepal (categorized as U5MR between 30 and 60 per 1000 live births) and WHO cut-off criteria of 70/1000 live births (categorized as U5MR between 60 and 90 per 1000 live births). We further included two categories of U5MR (between 90 and 120) and the high mortality category with U5MR greater than 120.

**Health service delivery**

- Sub-group analysis by measles immunization and Vitamin A coverage: Since not many trials reported the background level of immunization coverage, we referred to other national or state-level estimated data. We conducted this analysis following the cut-off criteria by the WHO for these indicators (WHO, 1998).

**Child nutrition status**

- Since not all trials reported data on baseline/background VAD and night, we were not able to conduct the subgroup analysis by such. We performed subgroup analysis by wasting level reported in the trials

### Sensitivity analyses

Some studies were at high risk of bias for sequence generation and incomplete outcome data. We conducted sensitivity analysis by excluding these studies at high risk of bias. We also considered studies with unclear allocation concealment and assessed their influence on the results. Further, given the heterogeneity, we repeated the analysis using a random-effects model. Since the analysis involved more than 10 studies, we plotted a funnel plot to examine small study bias.

### Summary of findings table

The quality of the evidence generated from the review was assessed using the approach, Grading of Recommendations Assessment, Development and Evaluation (GRADE) (Guyatt et al., 2011). We judged the quality of the evidence by examining it against different criteria. Criteria included overall risk of bias assessment, inconsistency, imprecision, indirectness of results and other considerations (study design limitations, publication bias). Once the judgements were made based on these criteria, the quality of the evidence for the outcome of mortality was judged either as having the high quality or moderate or low quality or as having very low quality. The Summary of findings for the main comparison table includes the results, i.e. effect estimates (relative and absolute risks), quality grading of the evidence with reasons (presented in the table footnotes). In addition, we applied the evidence obtained from the meta-analysis to the current U5MR in Nepal to show the likely impact of VAS in the present scenario. We also applied this estimate to the subnational level of U5MR considering one deprived and one least deprived evidence.

**References**

BEATON, G., MARTORELL, R., ARONSON, K., EDMONSTON, B., MCCABE, G. & ROSS, A. 1993. Effectiveness of vitamin A supplementation in the control of young child morbidity and mortality in developing countries–Nutrition policy discussion paper No. 13. Geneva: United Nations, Administrative Committee on Coordination/Subcommittee on Nutrition (ACC/SCN); 1993. 20.

COVIDENCE. 2021. *Covidence systematic review software* [Online]. Melbourne, Australia: Veritas Health Innovation. Available: <www.covidence.org> [Accessed 2021].

CRD 2009. *CRD's guidance for undertaking reviews in healthcare*, York Publ. Services.

DEEKS, J. J., HIGGINS, J. P. & ALTMAN, D. G. 2020. Analysing data and undertaking meta-analyses. *In:* J.P. HIGGINS, J. T., J. CHANDLER, M. CUMPSTON, T. LI, M.J. (ed.) *Cochrane Handbook for Systematic Reviews of Interventions.* Page and V.A. Welch.

GUYATT, G., OXMAN, A. D., AKL, E. A., KUNZ, R., VIST, G., BROZEK, J., NORRIS, S., FALCK-YTTER, Y., GLASZIOU, P. & DEBEER, H. 2011. GRADE guidelines: 1. Introduction—GRADE evidence profiles and summary of findings tables. *Journal of clinical epidemiology,* 64**,** 383-394.

HIGGINS, J., ELDRIDGE, S. & LI, T. 2021. Chapter 23: Including variants on randomized trials. *In:* HIGGINS, J., THOMAS, J., CHANDLER, J., CUMPSTON, M., LI, T., PAGE, M. & WELCH, V. (eds.) *Cochrane Handbook for Systematic Reviews of Interventions version 6.2.*

HIGGINS, J. P., ALTMAN, D. G., GØTZSCHE, P. C., JÜNI, P., MOHER, D., OXMAN, A. D., SAVOVIĆ, J., SCHULZ, K. F., WEEKS, L. & STERNE, J. A. 2011. The Cochrane Collaboration’s tool for assessing risk of bias in randomised trials. *Bmj,* 343**,** d5928.

REVIEW MANAGER 2020. Review Manager (RevMan) [Computer program]. Version 5.4. *Review Manager 5 (RevMan 5).* 5.4 ed.: The Cochrane Collaboration.

WHO. 1998. *Indicators for assessing vitamin A deficiency and their application in monitoring and evaluating intervention programmes* [Online]. World Health Organization. Available: <https://www.who.int/nutrition/publications/micronutrients/vitamin_a_deficiency/WHONUT96.10.pdf?ua=1> [Accessed 20 September 2020].

WHO. 2021. *Global Health Observatory data repository* [Online]. Geneva, Switzerland: World Health Organization. Available: <https://apps.who.int/gho/data/view.main.1600> [Accessed 20 January 2021].

WORLD BANK. 2021. *World Bank Open Data* [Online]. World Bank. Available: <https://data.worldbank.org/> [Accessed 20 January 2021].
