# Supplementary material for: Is routine Vitamin A supplementation still justified for children in Nepal? Trial synthesis findings applied to Nepal national mortality estimates
Source: PLoS One. 2022 May 18;17(5):e0268507. doi: 10.1371/journal.pone.0268507 (PMC9116662; doi:10.1371/journal.pone.0268507)
Supplement: S2 Appendix — (DOCX) [file pone.0268507.s009.docx]

## Characteristics of excluded studies

## Andersen 2018

| **Reason for exclusion** | Ineligible study design-not a randomized controlled trial. |
| --- | --- |

## Barreto 1994

| **Reason for exclusion** | Inappropriate outcome: Did not explicitly report child mortality. |
| --- | --- |

## Dibley 1996

| **Reason for exclusion** | Inappropriate outcome: Did not explicitly report child mortality. |
| --- | --- |

## Lin 2008

| **Reason for exclusion** | Inappropriate outcome: Did not explicitly report child mortality. |
| --- | --- |

## Chowdhury, 2002

| **Reason for exclusion** | Initially considered for inclusion but did not report the methods well and details were insufficient to be sure this was a genuine RCT. We tried to obtain additional information but were unsuccessful, so excluded this from the analysis. |
| --- | --- |
