# Supplementary material for: Is routine Vitamin A supplementation still justified for children in Nepal? Trial synthesis findings applied to Nepal national mortality estimates
Source: PLoS One. 2022 May 18;17(5):e0268507. doi: 10.1371/journal.pone.0268507 (PMC9116662; doi:10.1371/journal.pone.0268507)
Supplement: S3 Appendix. Characteristics of included studies — (DOCX) [file pone.0268507.s010.docx]

## Characteristics of included studies

## Agarwal 1995

| **Methods** | **Study design:** Cluster RCT was conducted in a rural part of Varanasi district in Uttar Pradesh, India  **Unit of randomization:** subcenter. A total of 16 clusters (subcentres) were randomly selected and divided into 4 subdivisions (4 subcentres in each), with drugs A (vitamin A) and B (placebo) distributed in 2 each randomly. At the end of the study, investigators found that vitamin A was distributed in 3 subdivisions (12 subcentres) and placebo in 1 only (4 subcentres) by mistake. |
| --- | --- |
| **Participants** | **Eligibility:** Children 1-72 months were included in the trial. A total of 17,778 children were approached but only 15,247 children were included in the final analysis based on the fact that they received at least 1 dose of vitamin A.  **Exclusion:** Children who were having established xerophthalmia at the start in both groups, were given one mega dose (200,000 IU.) of vitamin A and were excluded from the drug trial.  **Follow-up:** 12 months |
| **Interventions** | **Intervention:** Experimental group received 50,000 IU vitamin A plus 10 IU vitamin E if the child is 1-6 months and 100,000 IU vitamin A plus 20 IU vitamin E if the child is 7-72 months, every 4 months for 12 months.  **Comparison:** The corresponding control group received 10 (1-6 months) and 20 I.U.(7-72 months) of vitamin E, respectively per visit. |
| **Outcomes** | All-cause and cause-specific mortality due to diarrhoea, pneumonia, measles, and meningitis |
| **Other information** | **Baseline data** **U6MR/1000 children:** The background Under six mortality rates for children below 6 years of age were 27.7 and 23.3 per 1000 for the intervention and control groups, respectively, with significant differences in the 2 groups (P < 0.01) in the trial. However, as per National Family Health Survey (NFHS) 1992-93, U5MR in India was 109/1000 live births and Infant Mortality Rate was 78.5/1000 live births.  **Measles Mortality Rate**  4/3503 in the treatment group and 2/3937 in the control group  **Diarrheal Mortality Rate:**  14/3503 in the treatment group and 11/3937 in the control group.  **Child nutrition status**  **Nutritional status of children (in the trial)**  Underweight: Nutritional status graded based on wt-for-age:  Severe malnutrition (GRADE III): 10.5% (8111) in the experimental group and 6.9% (4763) in the control group at the beginning of the study and 9.5%(4153) in the experimental group and 7.2% (2824) at the end of the study.  Moderate malnutrition (GRADE II):44.4 % (8111) in the treatment group and 42.6% (4763) in the control group at the beginning of the study and 52.2%(4153) in the treatment group and 53.2% (2824) in the control group at the end of the study  **National data:**  While the state-level data for Uttar Pradesh for stunting was 59.5% and the national level figure stood at 52% as per NFHS 1992-93. Likewise, the state-level prevalence of wasting in Uttar Pradesh was 16% while the national level prevalence was 17.5%  **Xerophthalmia:** 363 and 127 children at baseline had xerophthalmia in the treatment and control group respectively who were excluded.  **Health service delivery**  National measles immunisation coverage as per NFHS 1992-93 was 42% while the immunisation coverage in Uttar Pradesh was 26.3%. No data on Vitamin A coverage was available. |
| **Notes** | The total study was planned in two phases. The first phase (January 90-March 91) expanded over 15 months (3 months for initial registration, 12 months for periodic intervention and subsequent mortality measurement to incorporate seasonal effects, if any). The second phase was a one-time measurement and was done exactly after one year after withdrawal (January 1991 to March 1992) of intervention on a subsample (on 1314 and 1200 children of the same age in the experimental and control groups respectively) |

#### **Risk of bias table**

| **Bias** | **Authors' judgement** | **Support for judgement** |
| --- | --- | --- |
| Random sequence generation (selection bias) | Unclear risk | Quote: "Out of the total 43 subcentres, 16 were randomly selected, four subdivisions (4 subcentres in each) were made and drugs A and B were distributed in two each randomly"Authors do not specify the methods of sequence generation  **Comment:** No additional information was provided. |
| Allocation concealment (selection bias) | Unclear risk | Quote: “Instead of subcentre if randomization was done at the individual level, there would have been a risk for interchange of the drug.  **Comment:** Authors do not provide sufficient information |
| Blinding of participants and personnel (performance bias) | Unclear risk | **Comment:** No information was provided by the authors. |
| Blinding of outcome assessment (detection bias) | Unclear risk | **Comment:** No information was provided by the authors. |
| Incomplete outcome data (attrition bias) | Unclear risk | **Comment:** No information was provided by the authors. |
| Selective reporting (reporting bias) | Unclear risk | **Comment:** No sufficient information was provided to allow for judgements |
| Recruitment bias | Unclear risk | Quote: "Out of a total of 43 subcentres, 16 were randomly selected, four subdivisions (4 subcentres in each) were made and drugs A and B distributed in two each randomly."  **Comment:** No information on whether the participants were identified and recruited before or after the randomization of clusters and no information on how the children were allocated to four subcentres with two subcentres receiving Vitamin A and two receiving placebo. |
| Baseline imbalance | High risk | **Comment:** Differential baseline mortality in children under six years of age.28/1000 children in the experimental group and 23/1000 in the control group. |
| Loss of clusters | Unclear risk | **Comment:** No sufficient information was provided to allow for judgement. |
| Incorrect analysis | Unclear risk | **Comment:** No adjustment for clustering was mentioned. Insufficient information to permit judgement. |
| Comparability with RCTs randomizing participants | Low risk | Comment: The effect of Vitamin A in cluster RCT is unlikely to be different than the effect in RCT |
| Other bias | Unclear risk | Comment: 11139 and 6639 children under six years of children were registered initially in the experimental and control group but 9987 and 5260 in the treatment and control group who received at least one dose were included in the analysis. No information about who refused to participate and who received all the doses and who received none. |

### Ben 1997

| **Methods** | A randomised, double-masked, placebo-controlled trial was conducted during 1993–95 in Belem and Mindara, two districts in Bissau, the capital of Guinea-Bissau, West Africa. |
| --- | --- |
| **Participants** | Eligibility: infants aged 6-9 months were eligible for inclusion in the trial.  Exclusion: Those with signs of xerophthalmia, history of previous vitamin A supplementation, history of measles infection before 9 months of age, or who had a positive haemagglutinin-inhibition assay (HIA) titre at 9 months of age were excluded. All infants reported to have had measles at 9-18 months of age were also excluded.  Sample: a total of 462 infants were randomised to either intervention or control group. |
| **Interventions** | There were 3 study groups: 1. Group I: included "infants aged 6 months and were randomly allocated to receive either a dose of measles vaccine at 6 months and a dose of measles vaccine at 9 months together with vitamin A supplement or the same dosing of measles vaccine with placebo as the supplement" 2. Group II: consisted of "infants who were randomly allocated either poliomyelitis vaccine at 6 months and a single dose of measles vaccine at 9 months with vitamin A supplement or the same vaccine doings with a placebo as the supplement" 3. Group III: included "infants who were older than 7·5 months at the beginning of the study or who were not found at home until they reached the age of 7·5 months, were included in the study at age 9 months and received a measles vaccine plus vitamin A or placebo supplement at that age" Vitamin A was supplemented in a single dose of 100,000 IU dissolved in 1 mL of vegetable oil along with 40 IU of vitamin E. Placebo: The placebo was 40 IU of vitamin E dissolved in 1 mL of vegetable oil. |
| **Outcomes** | Antibody response to the measles vaccine, all-cause mortality, the incidence of measles |
| **Other information** | **Child mortality and morbidity status**  The U5MR in the country in the year 1994 was 203/1000 live births as per the estimates developed by the UN Inter-agency Group for Child Mortality Estimation ( UNICEF, WHO, World Bank, UN DESA Population Division ) at [childmortality.org.](http://www.childmortality.org/) Likewise, IMR during the year 1994 stood at 121/1000 live births.  An investigation of child mortality in a semi-urban community, carried out in Guinea Bissau from April 1987 to March 1990 found persistent and acute diarrhoea as the most frequent causes of death, accounting for 43 and 31 deaths per 1000 children, respectively (Mølbak et al., 1992).  **Child Nutrition Status**  Guinea-Bissau is not a country known to have vitamin A deficiency, and with the abundance of palm oil and mangos, severe vitamin A deficiency among the mothers is unlikely. The Multiple Indicator Cluster Survey 2000 conducted in Guinea-Bissau in 2000 indicated:  Under-5's suffering from stunting, height for age indicator: 30% (MICS, 2000)  Under-5's suffering from moderate & severe wasting, weight for height indicator: 10%  **Health service delivery**  The Multiple Indicator Cluster Survey 2000 conducted in Guinea-Bissau in 2000 indicated:  Measles immunisation coverage: 48%  Vitamin A supplementation coverage:28% |
| **Notes** | The primary objective of the study was to calculate the antibody response to the measles vaccine when given vitamin A. The results for antibody response to the measles vaccine showed no significant difference between the groups. The study concluded that simultaneous administration of the measles vaccine and vitamin A has no negative effect on measles immunity. Vitamin A or placebo was given only at 9 months of age in all 3 study groups. We included all the numbers for all 3 intervention and placebo groups to report the outcomes of interest. |

#### **Risk of bias table**

| **Bias** | **Authors' judgement** | **Support for judgement** |
| --- | --- | --- |
| Random sequence generation (selection bias) | Low risk | Quote: "The allocation sequence was computer-generated." |
| Allocation concealment (selection bias) | Low risk | Quote: "The allocation sequence was kept in sealed envelopes and only released when all clinical laboratory analyses were completed." |
| Blinding of participants and personnel (performance bias) | Low risk | Quote: ". . .because of the young age of the participants, any difference in taste was irrelevant . . .None of the staff involved knew whether the bottles contained vitamin A or placebo . . ."  **Comment:** Probably done. |
| Blinding of outcome assessment (detection bias) | Low risk | Quote: "None of the staff involved knew whether the bottles contained vitamin A or placebo . . ." **Comment:** masking of treatment group assignment and treatment to study personnel likely to have been maintained throughout. |
| Incomplete outcome data (attrition bias) | Low risk | **Comment:** number lost to follow-up and those excluded were explicitly described and equal in both the groups. Loss to follow-up exceeded the number of deaths and children with measles. Reasons for missing data (migration) probably unrelated to treatment |
| Selective reporting (reporting bias) | Low risk | **Comment:** Deaths and prevalence of measles reported |
| Other bias | Unclear risk | **Comment:** authors report an imbalance in self-reported disease in the children aged 6 months at baseline. It is unclear how big an impact this will have had as the variable is not specific. |

### Daulaire 1992

| **Methods** | Study design: Cluster-randomized, non-placebo controlled trial conducted in Jumla district, Nepal |
| --- | --- |
| **Participants** | Sample: Infants and children aged 1-59 months in Jumla district of Nepal. 16 clusters were randomly assigned either to the vitamin A or control group. These included 7197 children, of which 3786 children were in the vitamin A group and 3411 were in the control group. |
| **Interventions** | In the experimental group, vitamin A was given in doses of 200,000 IU for children aged 12-59 months; 100,000 IU for children aged 6-12 months; and 50,000 IU for children aged < 6 months old. Vitamin A was supplemented once only and children were followed for 5 months. The Control group was given no supplementation. |
| **Outcomes** | All-cause mortality and cause-specific mortality due to diarrhoea, pneumonia, and measles |
| **Other information** | **Child mortality and morbidity rates:**  Background Infant mortality as reported in the trial was extremely high- 189 deaths per 1000 live births and a death rate in children aged 1-4 years of 52 per 1000 children per year. The demographic survey data indicated U5MR as 126/1000 live births  Diarrhoea and pneumonia were reported to be the leading causes of death in children after the first week of life. The trial also reported high seasonal variation in mortality was related to overall food deficit and the high prevalence of gastroenteritis and diarrhoea during the summer.  **Child nutrition status**  The trial reported substantial malnutrition (wasting) with 26% of children aged 1-4 years having arm circumferences. The level of stunting in the country during that period was reported to be 68% according to the Multiple Indicator Cluster Survey 1995.  Regarding the prevalence of xerophthalmia during that period, the trial referred to a survey of 3651 children under 5 years, which indicated the prevalence of active xerophthalmia to be 13-2%; xerophthalmia among infants was 1-5%, which is high for this age group.  ***Health service delivery***  Measles immunization coverage was around 37% in the country as per the estimates of World Bank data 1987. However, Vitamin A supplementation was not yet initiated in the country. |
| **Notes** | The study site was a remote, mountainous region of northwestern Nepal with a total population of about 80,000, with 12,000 children under 5 years of age. This area was considered one of the poorest and most medically underserved areas of the country. The infant mortality rate was 189 deaths per 1000 live births and child (1-4 years) mortality rate was 52 per 1000 per year. Malnutrition was prevalent in the study area, and 26% of children aged 1-4 years were suffering from substantial malnutrition. A survey of 3651 children under 5 years of age showed active xerophthalmia in 1.3% to 2% of the population and 1% to 5% among infants, which is high for this age group. Disaggregated data on mortality were available according to different age groups.2% of those who received vitamin A supplementation were reported to have experienced vomiting, fever, or diarrhoea within a few days after receiving vitamin A. |

#### **Risk of bias table**

| **Bias** | **Authors' judgement** | **Support for judgement** |
| --- | --- | --- |
| Random sequence generation (selection bias) | Low risk | Quote: "We randomly selected by card eight of the 16 sub-districts for vitamin A supplementation." **Comment:** probably done |
| Allocation concealment (selection bias) | Unclear risk | **Comment:** No sufficient information to permit judgement. |
| Blinding of participants and personnel (performance bias) | High risk | Quote: "There was no placebo or blinding." |
| Blinding of outcome assessment (detection bias) | High risk | Quote: "There was no placebo or blinding." |
| Incomplete outcome data (attrition bias) | Low risk | **Comment:** there was no loss to follow-up; coverage of intervention described in detail |
| Selective reporting (reporting bias) | Unclear risk | **Comment:** insufficient information to permit judgment |
| Recruitment bias | Unclear risk | **Comment:** No information about how the households were selected in the selected wards. |
| Baseline imbalance | Low risk | **Comment:** Tables I and II show a similar % of baseline characteristics in control and intervention |
| Loss of clusters | Unclear risk | Comment: No information about the loss of clusters |
| Incorrect analysis | Low risk | Quote: "The point estimates and 95% confidence intervals for the relative risk of mortality for vitamin A supplemented versus unsupplemented children were obtained from a Poisson regression analysis of deaths and aggregate person-years at risk in each subdistrict, incorporating variance overdispersion. This model, which uses sub-district as the unit of analysis, takes account of clustering due to randomization by sub-district rather than by child; the effect of clustering is reflected in an overdispersion parameter, which increases the width of the usual Poisson confidence intervals. The effect of clustering on the analysis was to increase the width of the confidence interval by 19% over the usual Poisson confidence interval".  **Comment:** Probably done |
| Comparability with RCTs randomizing participants | Low risk | **Comment:** As CRTs allow both the direct and indirect effects of an intervention to be captured, the effect of Vitamin A in cluster RCT is unlikely to be different than the effect in RCT. |
| Other bias | Low risk | **Comment:** No other bias |

### DEVTA trial 2013

| **Methods** | **Study design:** Factorial design, a cluster-randomised trial conducted in the defined catchment areas of 8338 state-staffed village child-care centres in 72 administrative blocks based in Lucknow, Uttar Pradesh, Northern India. The study was carried out for 5 calendar years between May 1999, and April 2004. |
| --- | --- |
| **Participants** | **Sample:**72 mainly rural administrative blocks were included of which 36 clusters received vitamin A supplementation and 36 acted as open control. A typical block included 10,000-20,000 children, covering -1 million children at any one time.  **Eligibility:** Children of age group 1-6 years were considered eligible for inclusion in the review |
| **Interventions** | Intervention: Children in the experimental group received 200 000 IU retinyl acetate in oil every 6 months for 5 years. Vitamin A was supplemented by village child care workers on mass-treatment days. The capsules were cut and poured into the child’s mouth. The study included:  1. usual care  2. 6-monthly vitamin A  3. 6-monthly albendazole  4. Both (Vitamin A and albendazole)  Comparison: Usual care-the control group did not receive any intervention (  no placebos were used) |
| **Outcomes** | All-cause mortality; cause-specific mortality due to diarrhoea, pneumonia, measles, and malnutrition; mean vitamin A serum levels; the prevalence of Bitot's spots, and measles and pneumonia morbidity |
| **Other information** | **Child mortality and morbidity rates:**  The total population at ages 1-6.0 years of these study areas is about one million, the 5-year probability of death at these ages is about 2-3% (Supplementary appendix). The state-level U5MR as reported in NFHS 2005-06 was 96/1000 live births. Likewise, IMR as reported in the trial was 87/1000 live births. According to a nationally representative survey NFHS 2005-2006, 9% of children were suffering from diarrhoea in the last 2 weeks.  **Child nutrition status:**  Prevalence of Bitot’s spot or night blindness was on average 2.5% at baseline in the study area during baseline. The state-level prevalence of stunting and wasting was 57% and 15% respectively according to NFHS 2005-06. While the prevalence of underweight was 42%.  ***Health service delivery***  Measles immunization coverage for Uttar Pradesh was 38% and Vitamin A supplementation coverage was around 6% according to NFHS 2005-6. |
| **Notes** | The study utilised the infrastructure of the Integrated Child Development Services (ICDS), which maintains child care centres called Anganwadi child care (AWC) centres across the state. Typical rural AWC employs an ICDS-funded AWC childcare worker who serves a village with a population of about 1000 (with 10–15% aged 1–6 years); the number of AWCs depends on the size of the village as large villages can have more AWCs. AWCs register about two-thirds of the children of age 1–6 years (and one-third of 5-year-olds) for possible nutritional supplementation. Another intervention considered in this study was albendazole for deworming. The study was approved by King George's Medical University. Surveillance for disease outcomes was done every 6 months, and children were not selected randomly for that but chosen from AWC lists. Deaths were recorded by 18 full-time, motorcycle village-to-village monitors. |

#### **Risk of bias table**

| **Bias** | **Authors' judgement** | **Support for judgement** |
| --- | --- | --- |
| Random sequence generation (selection bias) | Low risk | Quote: Neighbouring blocks (clusters), in groups of four, were randomly allocated in Oxford, UK, using a factorial design. Apart from the district, each block was in, no relevant details of it were known to those generating the random allocation.”  **Comment:** Probably done |
| Allocation concealment (selection bias) | Low risk | Quote: "Apart from the district each block was in, no relevant details of it were known to those generating the random allocation"  **Comment:** Probably done |
| Blinding of participants and personnel (performance bias) | High risk | **Comment:** Since Vitamin A was given on mass-treatment days by village health workers (and no placebo tablets are used, participants/ village health workers most likely were not blinded to treatment allocation |
| Blinding of outcome assessment (detection bias) | High risk | **Comment:** It seems that the outcomes assessors were aware of the treatment allocation and control, as parents were asked if their children received intervention on mass treatment days. |
| Incomplete outcome data (attrition bias) | Low risk | **Comment:** 91 AWCs (2%) lost to follow-up in the Vitamin A group and 82 AWCs (2%) lost to follow-up in the control group. |
| Selective reporting (reporting bias) | Low risk | **Comment:** The trial was registered at ClinicalTrials.gov, NCT00222547 and pre-specified outcomes were indicated in the protocol and analysed accordingly. |
| Recruitment bias | Low risk | Quote: “Neighbouring blocks (clusters), in groups of four (where possible in the same district), were randomly allocated in Oxford, UK, using a factorial design. it was determined before randomisation which AWCs were then functional, and hence potential study areas; |
| Baseline imbalance | Low risk | Quote: “The unit of randomisation in this cluster-randomised trial was the block. Neighbouring blocks (clusters), in groups of four (where possible in the same district), were randomly allocated. 36 blocks with (4233 AWCs functional ) were cluster-randomly allocated retinol supplementation every 6 months for 5 years and 36 open control (4278 AWCs functional)” |
| Loss of clusters | Unclear risk | **Comment:** No information was provided to form a judgement. |
| Incorrect analysis | Low risk | Quote: “The mean number of infant deaths per AWC was 117 (SD 1.8), the mean number of child deaths per AWC was 3.1 (SD 0・7), and there was a strong (0・69) correlation between the numbers of infant and child deaths per AWC in different blocks. Because any trial treatment in infancy began at around 9 months of age and most infant deaths occur much earlier, infant mortality cannot have been materially affected by it. Hence, this strong correlation reflects differences between low-risk and high-risk blocks that have nothing to do with trial treatment and that substantially affect both infant and child mortality (confirming this conclusion, the correlation between infant and child mortality was equally strong among blocks that had all had the same treatment. We, therefore, used the number of infant deaths as an explanatory factor to reduce chance variation in our main analyses of the effects of treatment allocation on the number of child deaths.”Sensitivity analyses explored the relevance of not adjusting for infant mortality or of additional adjustment for the district (by which randomisation had been stratified).  **Comment:** Cluster adjustment probably done. |
| Comparability with RCTs randomizing participants | Low risk | **Comment:** As CRTs allow both the direct and indirect effects of an intervention to be captured, the effect of Vitamin A in cluster RCT is unlikely to be different than the effect in RCT. |
| Other bias | High risk | **Comment:** there are concerns that surveillance for implementation of intervention and assessment of outcomes is not rigorous  . |

### Donnen 1998

| **Methods** | **Study design:** Individual randomised controlled trial conducted between April 1991 and May 1992 in south Kivu Province of Eastern Zaire, Congo |
| --- | --- |
| **Participants** | **Eligibility: C**hildren aged 0-72 months were eligible for inclusion in the trial and were included in the trial consecutively following their discharge from Lwiro  pediatric hospital and were followed for a year.  **Sample:** 358 children were randomly assigned to three groups: Vitamin A group (n=118)  Mebendazole (n=123) and Control group (n=117). |
| **Interventions** | **Vitamin A group:** Children > 12 months received 60 mg of Vitamin A (oily solution of retinyl palmitate) and children <12 months received 30 mg of Vitamin at the start of the trial and after a 6-months follow up.  **Mebendazole group:** Received 500mg of mebendazole at the start of the trial and every 3 months until 1 year.  **Control group:** did not receive any supplementation |
| **Outcomes** | Growth, mortality |
| **Other information** | **Child mortality and morbidity rates:**  The mortality rate of preschool children was high in outpatients (45/1000 per year) and hospitalised children. However, the national under-five mortality rate for Congo stood at 186 per 1000 live births in the year 1990 according to the World Bank estimates  **Child nutrition status:**  The prevalence of stunting across the study groups was 65.1% on average at baseline and the prevalence of wasting was 6.13% on average. The baseline prevalence of Vitamin A deficiency was 23.2% on average at baseline  **Health service delivery:**  Measles immunization coverage was 38% in the year 1990 according to World Bank data estimates. No Vitamin A supplementation program existed in the study area before the start of the trial. |
| **Notes** | Overall, 6% of children were lost to follow-up, with approximately equal proportions from each group.  5.4% of the children died in the Vitamin A group. 7.9% in the mebendazole group and 9.0% in the control group during the 1-y follow-up period. |

#### **Risk of bias table**

| **Bias** | **Authors' judgement** | **Support for judgement** |
| --- | --- | --- |
| Random sequence generation (selection bias) | Unclear risk | Quote: "As soon as the children were discharged from the hospital, they were randomly assigned to one of the three groups"  Comment: No further details are provided to make a judgment. |
| Allocation concealment (selection bias) | Unclear risk | Comment: No sufficient information was provided to form a judgement. |
| Blinding of participants and personnel (performance bias) | Unclear risk | Comment: No sufficient information was provided to form a judgement. |
| Blinding of outcome assessment (detection bias) | Unclear risk | Comment: No sufficient information was provided to form a judgement. |
| Incomplete outcome data (attrition bias) | Low risk | Quote: Overall, 6% of children were lost to follow-up, with approximately equal proportions from each group."  Comment: Number of children who died per group indicated as percentage and loss to follow-up per group not discussed in detail |
| Selective reporting (reporting bias) | Unclear risk | Comment: No sufficient information was provided to form a judgement. |
| Other bias | Low risk | Comment: No other concerns for bias. |

### Fisker 2014

| **Methods** | Study design: Individually-randomized, double-blind trial conducted in Guinea-Bissau |
| --- | --- |
| **Participants** | Eligibility: children aged 6-23 months were included. Exclusion criteria were vitamin A supplementation within the preceding month, and participation in another trial of 7587 children(VAS: 3787, placebo: 3800) was enrolled at vaccination contacts in Guinea-Bissau. |
| **Interventions** | Experimental group: Vitamin A was given in an amount of 100,000 IU for children aged 6-11 months and 200,000 IU for children aged 12-23 months. Control group: placebo was given in the same liquid amount as that in the intervention group. Supplementation was given at the time of vaccination. The vitamin A bottles contained vegetable oil with 200,000 IU vitamin A as retinyl palmitate and 40 IU vitamin E per mL oil; placebo bottles contained only 40 IU vitamin E per mL oil. |
| **Outcomes** | All-cause mortality, sex-specific mortality, diarrhoea incidence, respiratory infection, adverse events |
| **Other information** | **Child mortality and morbidity rates:**  Before the trial was started, the mortality rate was 23.8/1000 person-years of observation (PYRS) among children aged 6 to 59 months. During the study period, the U5MR level in the country was 113/1000 live births and IMR was 72/1000 as per 2010 World Bank data estimates.  On average 9.5% of the children had diarrhoea during the study period.  **Child nutrition status:**  The prevalence of Vitamin A deficiency was high across the study groups. The prevalence on average was 65.5% across the study area at baseline. The 2014 Multi-Indicator Cluster Survey indicated the prevalence of stunting at 28% and wasting at 6%.  **Health service delivery:**  Measles immunization coverage across the study groups was 41.5% on average. Vitamin A supplementation coverage before the enrolment into the study was about 54.5% |
| **Notes** | Adverse effects: (n=1756) Vitamin A=878 Placebo 878. VAS had no overall effect on clinical symptoms associated with increased ICP(Relative Risk(RR) = 1.07 (95%CI: 0.85–1.35)). The clinical signs associated with increased ICP were bulging fontanel, vomiting. Overall, VAS was not associated with having one or more symptoms associated with increased ICP (RR = 1.07 (95%CI: 0.85 1.35)).However, boys who received VAS had a higher risk (RR = 1.38(1.00–1.91)) whereas girls did not (RR = 0.81 (0.57–1.14)), resulting in a statistically significant interaction between VAS and sex(p = 0.03) |

#### **Risk of bias table**

| **Bias** | **Authors' judgement** | **Support for judgement** |
| --- | --- | --- |
| Random sequence generation (selection bias) | Low risk | Quote: "Mothers drew a lot from an envelope containing twenty lots; ten for each treatment arm. The number on the lot indicated from which of the two numbered bottles the children would receive an oral supplement." **Comment:** probably done |
| Allocation concealment (selection bias) | Low risk | Quote: “Coded vitamin A and placebo supplements were prepared by Skanderborg Pharmacy, Denmark.”  **Comment:** Probably done. |
| Blinding of participants and personnel (performance bias) | Low risk | Quote: “The dark brown bottles contained 10 ml.’’  **Comment:** Probably done |
| Blinding of outcome assessment (detection bias) | Low risk | **Comment:** study investigators were not aware of allocation. |
| Incomplete outcome data (attrition bias) | Low risk | **Comment:** 27 loss to follow-up in vitamin A group and 21 in the placebo group. Reason for attrition was given, and they were similar in both groups. |
| Selective reporting (reporting bias) | Low risk | **Comment:** the trial was registered with the number NCT00514891. All a priori outcomes are reported. |
| Other bias | Low risk | **Comment:** No other bias was observed. |

### Herrera 1992

| **Methods** | Study design: Double blind placebo-controlled Cluster randomised trial conducted between June 1988, and December 1990, in five rural councils in northern Sudan between June 1988, and December 1990. |
| --- | --- |
| **Participants** | **Sample**: Randomisation was done by households. The study enrolled a total of 28,753 children, of whom 14,455 were in the vitamin A group and 14,298 were in the placebo group.  **Eligibility:** Children between 9 and 72 months of age were included in the study.  **Exclusion:** Children with xerophthalmia were excluded from the study. |
| **Interventions** | **Intervention:** 200,000 IU of retinol palmitate along with 40 IU of vitamin E were given to children in the Vitamin A group. The intervention was given every 6 months for 18 months.  Comparison: The comparison group (Placebo) received 40 IU of vitamin E only |
| **Outcomes** | All-cause mortality; cause-specific mortality due to diarrhoea, measles |
| **Other information** | **Child mortality and morbidity rates:**  **Baseline data:**   - 0.3% of children had measles in the seven days preceding the study in both treatment and control group.17.3% of children had diarrhoea in the seven days preceding the study across the study groups. - U5MR was 135/1000 live births and IMR was 77/1000 as per Sudan Demographic Health Survey 1989-90.   **Child nutrition status**   - VAD was present and there was local awareness of the problem - At baseline, the prevalence of xerophthalmia was 2.85% across the study groups. Likewise, the prevalence of stunting was 38% and wasting was around 6%. At baseline, the mean Vitamin A dietary intake was around 160.5 retinol equivalents.   ***Health service delivery***  Measles immunization coverage was 67% and Vitamin A supplementation coverage was 20.05% during the period as per Sudan DHS 1989-90. |
| **Notes** | Randomisation was done by households. |

#### **Risk of bias table**

| **Bias** | **Authors' judgement** | **Support for judgement** |
| --- | --- | --- |
| Random sequence generation (selection bias) | High risk | **Quote**: "Randomisation was done by household . . . Assignment to treatment group was achieved by the two interviewers visiting alternate households throughout the village. All eligible children in alternate households were assigned to receive, every 6 months, either a capsule of 60 mg (200 000 IU) of vitamin A and 40 mg (40 IU) of vitamin E or a capsule of 40 mg of vitamin E without vitamin A."  **Comment:** Random method probably not used. |
| Allocation concealment (selection bias) | Unclear risk | **Comment:** No sufficient information to form a judgement. |
| Blinding of participants and personnel (performance bias) | Low risk | **Quote**: "The capsules were colour-coded to avoid the possibility of mix-ups, but none of the study team members was aware which was the experimental capsule and which was the placebo until the end of data collection. All eligible children in a household received capsules of the same colour."  **Comment:** Probably done |
| Blinding of outcome assessment (detection bias) | Low risk | Quote: "Only the manufacturer knew the contents of the capsules until after data collection and preliminary analysis of the results."  **Comment:** probably done |
| Incomplete outcome data (attrition bias) | Low risk | Quote: 3320 children did not receive 1 or 2 of the 3 vitamin A or placebo capsules. Most of this non-compliant group consisted of children absent from the household at the time of follow-up, whereas others had moved away or refused to take part further. As a group, the non-compliant children tended to be from poorer households than those who continued in the study. However, there were no significant differences between vitamin A and placebo groups in the number of non-compliant subjects or their ages, sex, or nutritional status. At the end of the study, the survival status was known for all children originally enrolled apart from those excluded because of xerophthalmia and 167 others who could not be traced."  **Comment:** Losses to follow up were not significantly different from those that remained in the study. |
| Selective reporting (reporting bias) | Unclear risk | **Comment:** No protocol or trial registration number has been referred. |
| Recruitment bias | Unclear risk | **Comment:** No sufficient information to allow for judgement. |
| Baseline imbalance | Low risk | Quote: “There were no important differences between Vitamin A and placebo groups in the rate of xerophthalmia, Vitamin A intake, age distribution or nutritional status." |
| Loss of clusters | Unclear risk | **Comment:** No sufficient information to allow for judgement. |
| Incorrect analysis | Low risk | Quote: "Treatment effects were calculated by use of Relative Risks(RR) with 95% confidence interval. Mortality rates were calculated for children at baseline and alternatively for those who receive a trial capsule in the previous round. Logistic regression and discrete-time survival analyses were used to estimate the overall treatment effect adjusted for baseline differences and to identify predictors of mortality, including dietary Vitamin A intake. Controlling for the geographic site, a round of observation, morbidity, anthropometry, water supply, poverty, treatment with Vitamin A and baseline differences between the two groups did not change the results."  **Comment:** Cluster adjustment probably done. |
| Comparability with RCTs randomizing participants | Low risk | **Comment:** The effect of Vitamin A in cluster RCT is unlikely to be different than the effect in RCT. |
| Other bias | Unclear risk | **Comment:** Insufficient details provided to permit judgement. |

### Pant 1996

| **Methods** | Study design: Cluster-randomised trial conducted in seven districts of the Terai and mid-hill areas rural Nepal |
| --- | --- |
| **Participants** | Sample: from 100 potentially eligible cluster sites, 75 were randomised (approximately 25,301 children). 457 subdistricts in 438 rural and 19 town locales. Designed to include about 450 children below the age of 11 years.  Eligibility: children aged 6 months to 10 years were eligible to participate in the study. |
| **Interventions** | Study included:  1. Group I: vitamin A was given as a single dose via a capsule (100,000 IU for children aged 6-12 months and 200,000 IU for children aged 1-10 years) 2. Group II: control (not described in detail) 3. Group III: nutritional education  Study duration: 24 months |
| **Outcomes** | All-cause mortality and Bitot's spots |
| **Other information** | **Child mortality and morbidity rates:**  U5MR of the country stood at 118/1000 live births during the period when the study was conducted according to Nepal Family Health Survey 1996. Likewise, IMR was around 79/1000 live births.  28% of children had diarrhoea two weeks before the survey.  **Child nutrition status:**  The background level of wasting reported in the trial was high (67%). However, the level of wasting in the country was 11.2%, followed by a high level of stunting (48%) and underweight (47%). The prevalence of night blindness among children aged 6-35 months was about 1% according to NFHS 1996.  **Health Service Delivery:**  Measles immunisation coverage was about 57% and Vitamin A supplementation coverage was about 32% in the country as per NFHS 1996. |
| **Notes** | No details on loss to follow-up were given. Inclusion/exclusion criteria were inadequately described. |

#### **Risk of bias table**

| **Bias** | **Authors' judgement** | **Support for judgement** |
| --- | --- | --- |
| Random sequence generation (selection bias) | Low risk | Quote: “Using random tables and the reference number for each block in the subdistrict we selected one block (or cluster of wards)from each subdistrict.”  Comment: probably done. |
| Allocation concealment (selection bias) | Unclear risk | **Comment:** insufficient detail provided to make a judgment |
| Blinding of participants and personnel (performance bias) | Unclear risk | **Comment:** insufficient detail provided to make a judgment |
| Blinding of outcome assessment (detection bias) | Unclear risk | **Comment:** insufficient detail provided to make a judgment |
| Incomplete outcome data (attrition bias) | Unclear risk | Quote: "A total of 75 of the 100 sites and 296 of the 395 wards were used in this analysis; no information given as regards how incomplete outcome data were addressed." |
| Selective reporting (reporting bias) | High risk | **Comment:** Selectively reported wasting, bitot’s spot and mortality. No information about the xerophthalmia and other nutrition status |
| Recruitment bias | Unclear risk | **Comment:** No information about how the households were selected in the selected wards |
| Baseline imbalance | Unclear risk | **Comment:** No information about baseline characteristics by control and intervention |
| Loss of clusters | Unclear risk | **Comment:** No information about the loss of clusters |
| Incorrect analysis | Low risk | Quote: "The logistic regression analysis was set up to identify those community, household and individual-level risk factors that could best be used to predict risk for all three of the health status parameters. A total of 48 predictors (independent variables) were used that covered the following: community development; the size of the ward (indicated in Table 2) community agriculture; history of disease epidemics within the previous 3 years; economics of the ward; availability of government development projects; household wealth; household sanitation; degree of participation in the vitamin A child survival project; and the nutritional status of the children."  **Comment:** Cluster adjustment probably done. |
| Comparability with RCTs randomizing participants | Low risk | **Comment:** The effect of Vitamin A in cluster RCT is unlikely to be different than the effect in RCT. |
| Other bias | Unclear risk | **Comment:** insufficient detail provided to make a judgment |

### Rahmathullah 1990

| **Methods** | Study design: Cluster Randomised Controlled Masked Clinical trial conducted in three drought-prone economically and environmentally deprived panchayat unions in the Trichy district of Tamil Nadu in Southern India. |
| --- | --- |
| **Participants** | Eligibility: Children of age 6-60 months were included in the study.  Sample: Three panchayat unions (local-government areas) were selected and the panchayat union was the clustering unit.206 clusters were formed and the majority of such clusters included 50-100 children of age 6-60 months. There were a total of 15,419 children with 7764 in the Vitamin A group and 7655 in the control group. |
| **Interventions** | Intervention: Children in the Vitamin A group received weekly doses of 8333 IU vitamin A and 20 mg vitamin E for 52 weeks and supplements were given to children by the community health volunteers. Children diagnosed with xerophthalmia at baseline, six months or final examination were given a high dose of Vitamin A (209µmol) and Vitamin E (46µmol) and were part of the study. Children who missed 7 consecutive dosages were excluded from the analysis.  Control: The control group received 20 mg of vitamin E dissolved in peanut oil. |
| **Outcomes** | All-cause mortality; cause-specific mortality due to diarrhoea, measles, and respiratory disease; incidence of diarrhoea and respiratory disease morbidity |
| **Other information** | **Child mortality and morbidity rates:**  Average U5MR in India during that time: 20/1000 (mentioned in the study). The World Bank data estimates the U5MR as 130/1000 live births for the year 1989.  IMR was reported as 79 per 1,000 live births in 1988-92 NFHS survey data. However, IMR was reported as 64/1000 live births(1988-99) in the trial  For the baseline, the equivalence of age sex, the incidence of diarrhoea and respiratory diseases, anthropometry, xerophthalmia, U5MR, household economic and hygiene status, and serum retinol level were mentioned but no data was shown. According to NFHS 1992-93, 10% of all under-five children were reported to be suffering from diarrhoea in the last 2 weeks.  **Child nutrition status**  Baseline data in the study population indicated the prevalence of stunting at 31%, wasting at 23%, and prevalence of both stunting and wasting at 18%. Likewise, the prevalence of xerophthalmia was high at 11% with night-blindness at 3% and Vitamin A Deficiency (Serum retinol concentration (≤0.70 µmol/l)=37% (n=280).  ***Health service delivery***  Measles immunization coverage as per World Ban data in the year 1989 was 42%.In the trial: only 1% of the children under 60 months in the study area received Vitamin A under the national Vitamin A program. |
| **Notes** | The study was conducted in a drought-prone area. Only 1% of the children under 60 months in the study area received Vitamin A under the national Vitamin A program. Children with xerophthalmia were treated with a large dose of Vitamin A (209µmol) and they continued to be part of the study |

#### **Risk of bias table**

| **Bias** | **Authors' judgement** | **Support for judgement** |
| --- | --- | --- |
| Random sequence generation (selection bias) | Unclear risk | Quote: "The clusters were arranged according to population size; after a random start, they were assigned alternately to the treated or control groups."  **Comment:** No information on the method used for sequence generation provided to allow for judgement |
| Allocation concealment (selection bias) | Low risk | Quote: ". no one associated with the study was aware of the colour code, which was held by the Hoffmann-LaRoche until the study ended."  **Comment:** Probably done |
| Blinding of participants and personnel (performance bias) | Low risk | Quote: "The appearance and taste of the solutions were identical . . . no on associated with the study was aware of the colour code, which was held by the Hoffmann-LaRoche until the study ended."  **Comment:** Probably done |
| Blinding of outcome assessment (detection bias) | Low risk | Quote: “Community volunteers knew that they were responsible for dispensing from one colour-coded bottle, but they were unaware of what it contained other than vitamins.” |
| Incomplete outcome data (attrition bias) | Unclear risk | Quote: "There was no difference in rates of contact between the treated and control groups. The reasons for lack of contact included moving from the study area . . ."  **Comment:** Although reasons for loss to follow-up were given with a note that there was no difference in contact rates between the 2 groups, no information on how many of the children were lost across each group |
| Selective reporting (reporting bias) | Low risk | **Comment:** all-important outcomes are given in results as mentioned in the Methods section. |
| Recruitment bias | Low risk | Quote: “From the 15,419 children identified and examined at baseline, 206 clusters were formed…The clusters were arranged according to population size; after a random start, they were assigned alternately to the treated or control groups.” |
| Baseline imbalance | Unclear risk | Quote: “The baseline data was checked for the following characteristics: age and sex distribution, 1-month history of diarrhoea and respiratory disease, anthropometric indexes of nutritional status, xerophthalmia status, 5-year retrospective history of mortality of children under 5, household economic, household hygienic status, and serum retinol levels. Matching was satisfactory at baseline for all the variables examined.”  **Comment:** Although the information about baseline balance between the groups was mentioned, data for baseline comparison was not provided. |
| Loss of clusters | Unclear risk | **Comment:** No information to permit judgement. |
| Incorrect analysis | Low risk | Quote:” Randomization according to cluster rather than according to child introduced a moderate increase (about 30%) in the variance of the estimators of the relative risk of death in the treated group as compared with the control group.”  **Comment:** Rise invariance (stated as 30%) due to cluster sampling recognized. |
| Comparability with RCTs randomizing participants | Low risk | The effect of Vitamin A in cluster RCT is unlikely to be different than the effect in RCT. |
| Other bias | Low risk | **Comment:** No other bias |

### Ross 1993 HEALTH

| **Methods** | Study design: Randomised, double-blind controlled trial conducted in Guinea savannah area of Ghana |
| --- | --- |
| **Participants** | 1455 children were included. Children aged 6-59 months were included. Those with active xerophthalmia or measles were excluded from the trial the moment they were confirmed. Children followed for an average of 9.8 months. |
| **Interventions** | Children in the vitamin A group received either 200,000 IU retinol equivalent for participants aged > 12 months or 100,000 IU for children aged 6-12 months. The control group received a placebo (peanut oil). Interventions were given every 4 months for 12 months. |
| **Outcomes** | All-cause mortality; mean daily prevalence of respiratory tract disease, diarrhoea, measles, malaria; mean vitamin A serum levels; all-cause hospitalisations |
| **Other information** | **Child mortality and morbidity rates**  U5MR in Ghana during the year 1990 was 127/1000 live births and IMR was 80/1000 live births as per World Bank data.  The prevalence of diarrhoea among the children was 20% according to Ghana Demographic Health Survey 1993.  **Child Nutrition status**  The baseline prevalence of xerophthalmia in the study area/population was 1.5%. Likewise, based on baseline serum retinol concentrations, 15.8% were severely deficient (<0.35µmol/l) and 57.6% moderately deficient (<0. 35-0.69µmol/l) in the study area. Baseline stunting was 48.2%, underweight was 41.2% and wasting was 4.2% in the study population. Similarly, the baseline Vitamin A deficiency was about 16%.  **Health service delivery:**  No Vitamin A supplementation program within the study area. Average measles immunisation coverage in the study groups at baseline was about 50%. |
| **Notes** | The study populations were rural and their main staple foods are deficient in carotenoids and vitamin A. Vitamin A deficiency and xerophthalmia were recognised as problems locally. Children were visited weekly for 1 year. |

#### **Risk of bias table**

| **Bias** | **Authors' judgement** | **Support for judgement** |
| --- | --- | --- |
| Random sequence generation (selection bias) | Unclear risk | Quote: "Randomisation was blocked in both studies to ensure similar numbers of children in each group in each part of the study area."  **Comment:** No sufficient information to permit judgement. |
| Allocation concealment (selection bias) | Low risk | Quote: "Randomisation was carried out in London by an independent statistician, who held the randomisation code and who also did an interim analysis of the mortality results from the Survival Study for the trial's data monitoring committee after a year of follow-up."  **Comment:** Probably done |
| Blinding of participants and personnel (performance bias) | Low risk | Quote: "Vitamin A and placebo were supplied by Hoffmann-La-Roche's Sight and Life Programme, and were similar in taste and colour. In the Survival Study, liquid vitamin A or placebo was supplied in opaque 150 mL bottles containing 20 IU/mL vitamin E alone (placebo) or plus 100,000 IU/mL retinol equivalent as retinyl palmitate (vitamin A) in purified peanut oil. Each bottle had a unique number, and was labelled with a cluster code before despatch to Ghana."  **Comment:** Probably done |
| Blinding of outcome assessment (detection bias) | Low risk | Probably done |
| Incomplete outcome data (attrition bias) | Unclear risk | **Comment:** morbidity information was missing for 5% to 7% of the weekly follow-up visits, owing to temporary absences of the study children or their mothers, but the missing data were equally distributed between the treatment groups. |
| Selective reporting (reporting bias) | Unclear risk | **Comment:** No protocol is available. |
| Other bias | Low risk | **Comment:** No other bias |

### Ross 1993 SURVIVAL

| **Methods** | A cluster-randomised trial was conducted in Ghana. The Survival Study trial was carried out between September 1989 and December 1991 |
| --- | --- |
| **Participants** | The study area is divided into 185 geographical areas. 92 clusters were assigned to Vitamin A and 93 for placebo treatment. The study involved 185 clusters that included 21,906 children. For survival study, children were followed for an average of 18.2 months.  Exclusion: Confirmed xerophthalmia cases were excluded. |
| **Interventions** | The experimental group received vitamin A supplementation in a dose of 100,000 IU for children aged 6-11 months and 200,000 IU for older children.  The comparison group received a placebo. Vitamin E in a dose of 20 IU was given to both groups. Interventions were delivered every 4 months for 24 months. |
| **Outcomes** | All-cause mortality and cause-specific mortality due to diarrhoea, respiratory disease, measles, and meningitis; mean vitamin A serum levels; malaria prevalence |
| **Other information** | **Child mortality and morbidity:**  U5MR in Ghana during the year 1989 was 132/1000 live births and IMR was 83/1000 live births as per World Bank data. Prevalence of diarrhoea among the children was 20% according to Ghana Demographic Health Survey 1993.  No Vitamin A supplementation program within the study area. Average measles immunisation coverage in the study groups at baseline was about 50%.  **Child nutrition status**  The baseline prevalence of Xerophthalmia was 0.7% in the study population. Likewise, the baseline serum retinol concentrations measurements indicated that 14.4% were severely deficient (<0.35µmol/l) and 42.5% were moderately deficient (<0. 35-0.69µmol/l). Similarly, the baseline Vitamin A deficiency was about 14%. The baseline prevalence of wasting was 43.5% in the study population. 43% of children had stunting and 7% had wasted as per World Bank data 1988.  ***Health service delivery***  No Vitamin A supplementation program within the study area. Average measles immunisation coverage in the study groups at baseline was about 44.5%. |

#### **Risk of bias table**

| **Bias** | **Authors' judgement** | **Support for judgement** |
| --- | --- | --- |
| Random sequence generation (selection bias) | Unclear risk | Quote: "Randomisation was blocked in both studies to ensure similar numbers of children in each group in each part of the study area." **Comment:** No additional information to permit judgement. |
| Allocation concealment (selection bias) | Low risk | Quote: "Randomisation was carried out in London by an independent statistician, who held the randomisation code and who also did an interim analysis of the mortality results from the Survival Study for the trial's data monitoring committee after a year of follow-up." **Comment:** code was protected for the duration of the trial. |
| Blinding of participants and personnel (performance bias) | Low risk | Quote: "Vitamin A and placebo were supplied by Hoffmann-La-Roche's Sight and Life Programme, and were similar in taste and colour. In the Survival Study, liquid vitamin A or placebo was supplied in opaque 150 mL bottles containing 20 IU/mL vitamin E alone (placebo) or plus 100,000 IU/mL retinol equivalent as retinyl palmitate (vitamin A) in purified peanut oil. Each bottle had a unique number, and was labelled with a cluster code before despatch to Ghana." **Comment:** probably done |
| Blinding of outcome assessment (detection bias) | Low risk | **Comment:** given the blinding procedures in place elsewhere in the study, this was probably adequate. |
| Incomplete outcome data (attrition bias) | Unclear risk | **Comment:** 8.4% (1847) of children lost to follow-up and similar between treatment groups. The reasons for losses to follow-up are not provided. |
| Selective reporting (reporting bias) | Unclear risk | **Comment:** No sufficient information to permit judgement. |
| Recruitment bias | Low risk | **Comment:** All children in the selected blocks were selected who are eligible. |
| Baseline imbalance | Unclear risk | **Comment:** There is no comparison information. |
| Loss of clusters | Unclear risk | **Comment:** No sufficient information was provided to permit judgement. |
| Incorrect analysis | Low risk | Quote: "The ratio of the mean mortality rate in the Vitamin A and placebo clusters was used to measure the mortality impact of supplementation with 95% CI calculated by the method of Armitage and Berry. Statistical Methods in Medical Research. The CIs for rate ratios were calculated by means of a formula for determining the CI of the ratio of two normal means."  **Comment:** Cluster adjustment probably done. |
| Comparability with RCTs randomizing participants | Low risk | The effect of Vitamin A in cluster RCT is unlikely to be different than the effect in RCT. |
| Other bias | Unclear risk | **Comment:** No sufficient information was provided to allow for judgement. |

### Sommer 1986

| **Methods** | Study design: Cluster-randomized trial conducted in a rural area of Indonesia, in Aceh Province, which is at the northern tip of Sumatra.  Study duration: 9-13 months |
| --- | --- |
| **Participants** | Sample: The sampling frame consisted of 2048 villages in Aceh, Utara and Pidie, two rural kabupatens (districts) where there had no current or planned development projects or vitamin A supplementation schemes. 450 villages were systematically selected for the study and were randomised into programme villages (n=229) or control villages (n=221). 29,236 children from 450 villages (cluster sites) were enumerated at baseline.  Eligibility: children aged 0-5 years were considered for inclusion in the study.  Exclusion: All children with active xerophthalmia at baseline examination received at least one large dose of vitamin A and were excluded from the analyses of subsequent morbidity and mortality. |
| **Interventions** | Intervention: Standard capsules (200 000 IU vitamin A) along with 40 IU vitamin E were given to every child aged 1-5 years in programme villages, by a local volunteer trained to do so. The first dose was given 1-3 months after the baseline examination and the second 6-8 months later.  Comparison: No treatment-Control group served as a waiting list control which was randomised after the follow-up examination. |
| **Outcomes** | All-cause mortality, xerophthalmia |
| **Other information** | **Child mortality and morbidity rates:**  U5MR as per Indonesia Demographic Health Survey 1981-86 was 115.5/1000 live births and IMR was 80/1000 live births. At baseline, around 22.3% of children in the study population had measles at any time in the past. Likewise, 7.9% of children in the study population had diarrhoea in the past seven days.  **Child nutrition status**  At baseline, the average prevalence of night-blindness in the study population was 1.21%, Bitot’s spots: 1.3%, Active xerophthalmia: 2.06%. Likewise, the level of stunting at baseline was on average 8.55% and wasting was 3.35%.  ***Health service delivery***  Measles immunization coverage was about 13%.No Vitamin A supplementation schemes were implemented in the study districts. |
| **Notes** | ICCs from analyses reported to have been adjusted for design effect. |

#### **Risk of bias table**

| **Bias** | **Authors' judgement** | **Support for judgement** |
| --- | --- | --- |
| Random sequence generation (selection bias) | Unclear risk | **Quote:** "From a random start, 450 villages were systematically selected for the study; these were then randomised for capsule distribution after the baseline examination . . ."  **Comment:** No sufficient information to permit judgement. |
| Allocation concealment (selection bias) | Unclear risk | **Comment:** No sufficient information to form a judgement. |
| Blinding of participants and personnel (performance bias) | Unclear risk | **Quote**: "The Government of Indonesia would not condone the use of placebos but field-workers collecting demographic data were unaware that mortality was a research issue."  **Comment:** Although the study has been described as a controlled study, there is no adequate description of what the control group received. |
| Blinding of outcome assessment (detection bias) | Unclear risk | Quote: "The Government of Indonesia would not condone the use of placebos but field-workers collecting demographic data were unaware that mortality was a research issue."  **Comment:** Although field-workers were unaware that mortality was a research issue, the inadequate description of what the control group received makes it unclear. |
| Incomplete outcome data (attrition bias) | Unclear risk | Quote: "Follow-up information was available on 89% of the programme children and 88.4% of the controls. The age and sex distribution of children lacking follow-up were identical in the two groups."  **Comment:** Authors, although have mentioned the indicated percentage remaining per group at follow-up and have mentioned age and sex among those lacking follow up was identical, no additional information was provided. |
| Selective reporting (reporting bias) | Unclear risk | **Comment:** trial protocol not available |
| Recruitment bias | Unclear risk | **Comment:** No sufficient information to permit judgement. |
| Baseline imbalance | Unclear risk | Quote: “The populations were similar in most baseline characteristics investigated except for xerophthalmia and history of recent diarrhoea, which was slightly more prevalent among the controls.”  Comment: Although it has been mentioned that the baseline characteristics were similar across the groups no data was provided for baseline demographic and socio-economic variables including the occupation of the head of the household, maternal education, source of drinking water, distance to the nearest elementary school, and distance to the nearest health centre. |
| Loss of clusters | Unclear risk | **Comment:** No information provided to form a judgement. |
| Incorrect analysis | Low risk | **Quote:** “Statistical tests for significance and development of confidence intervals were adjusted for clustering associated with randomization by village rather than by individual, and for the small number of events expected and observed in any one village by applying Poisson regression with extra-poisson variation to account for natural variability in mortality among villages.”  **Comment:** Cluster adjustment is probably done through adjustment of confidence intervals. |
| Comparability with RCTs randomizing participants | Low risk | The effect of Vitamin A in cluster RCT is unlikely to be different than the effect in RCT. |
| Other bias | Unclear risk | Comment: Insufficient information to form a judgement. |

### Venkata Rao 1996

| **Methods** | Study design: Individually Randomized double-blind placebo-controlled field trial conducted in Villupuram Health Unit District of Tamil Nadu, South India. The study was initiated in April 1991 and completed in May 1993. |
| --- | --- |
| **Participants** | Sample: 909 infants were randomised into three intervention groups. 50% of the infants in the study were males and 52% were exposed to passive smoking.  Eligibility: Infants aged six months. |
| **Interventions** | Intervention: Infants received 2 ml of syrup containing 200,000 IU of Vitamin A (as per the programme in Tamil Nadu). The study included three groups:  Group AA: Both mothers received and infants (311) received vitamin A  2. Group AP: mother received vitamin A while infant (301) received placebo  3. Group PP: both mother and infant (297) received a placebo  Placebo: 2ml of sesame oil |
| **Outcomes** | All-cause mortality and cause-specific mortality due to diarrhoea and respiratory disease. Incidence of diarrhoea and respiratory disease morbidity |
| **Other information** | **Child mortality and morbidity rates:**  U5MR was 109 per 1,000 live births and IMR was 78.5/1000 livebirths according to NFHS 1992-93. The incidence of diarrhoea (at least one episode) was greater than 90% in the study population.  10% of under-five children reported diarrhoea in the last two- weeks according to NFHS 1992-1993.  **Child nutrition status:**  The trial cited their unpublished study conducted on 1,620 children aged 5-10 years from a random sample of six Municipal schools showed the prevalence of Bitot spots to be 2.1%, and that of conjunctival xerosis to be 1.0%. The prevalence of stunting was 52% and wasting was 17.5% and underweight was 53% as per NFHS 1992-93.93). Likewise, the prevalence of underweight in Tamil Nadu was 48.2%  ***Health service delivery :***  In India, measles immunization coverage was 42% (NFHS 1992-93) and in Tamil Nadu was 72%. No information on routine/previous vitamin A distribution in the area. |
| **Notes** | Venkatarao 1996 studied the impact of 300, 000 IU vitamin A to the mother soon after delivery and to the infant (200, 000 IU) at six months on morbidity in infancy in a field trial in rural Tamil Nadu.  For the purpose of our study, we have considered AA as the Vitamin A group and (PP) as the control group. |

#### **Risk of bias table**

| **Bias** | **Authors' judgement** | **Support for judgement** |
| --- | --- | --- |
| Random sequence generation (selection bias) | Unclear risk | Quote: "Each pair of subjects enrolled for the study was randomly allocated to one of the following three groups: (i) AA-Both mother and infant received Vitamin A, the former soon after delivery and the latter at 6 months; (ii) AP: mother received Vitamin A but her infant received a placebo (Sesame oil); and (iii) PP: both mother and infant received placebo, the former Vitamin E and the latter  Sesame oil."  **Comment:** Insufficient information to allow judgement |
| Allocation concealment (selection bias) | Unclear risk | **Comment:** No information provided to form a judgement. |
| Blinding of participants and personnel (performance bias) | Low risk | Quote: "At the age of 6 to 6Vi months, the infant was weighed again and given the appropriate syrup by the Medical Officer from coded bottles, supplied again by the Statistical Section at the Camp Office."  **Comment:** Probably done |
| Blinding of outcome assessment (detection bias) | Low risk | Quote: “At the age of 6 to 6Vi months, the infant was weighed again and given the appropriate syrup by the Medical Officer from coded bottles, supplied again by the Statistical Section at the Camp Office… Data on morbidity was collected for each day until the child attained one year of age (together with immunization status and infant feeding practices), by field Investigators at home visits… The trial was conducted double-blind, and quality control of the morbidity data collected by the field investigators was undertaken throughout.”  **Comment:** Probably done |
| Incomplete outcome data (attrition bias) | Unclear risk | Quote: "4 each in the AA and AP groups and 5 in the PP group were withdrawn from the trial on medical grounds such as congenital abnormalities, epileptic fits or jaundice. Migration accounted for the loss of 34 infants in the AA group, 25 in the AP group and 20 in the PP group while 7, 9 and 7 were excluded due to other miscellaneous reasons. Of the remaining 263, 255 and 256 infants in the three groups, 233 in the AA and 228 each in the AP and PP groups were followed- up very regularly and form the basis for analyses in this report."  **Comment:** Although they have provided reasons for losses by groups. However, No reasons were provided for why only 233 in the AA and 228 each in the AP and PP groups were followed and retained in the analysis when there were 263, 255 and 256 infants in each of the groups after attrition. No information was provided on 30, 27 and 28 infants who were lost. |
| Selective reporting (reporting bias) | Unclear risk | **Comment:** No protocol or trial registration number has been referenced, so it is not clear if the outcomes are measured and reported |
| Other bias | Unclear risk | Quote: “The three groups (AA, AP and PP) had very similar distributions concerning all the above baseline characteristics.”  Comment: Although it has been mentioned that the baseline characteristics are similar, did not provide data comparisons across the groups to support the similarities between the groups. |

### Vijayaraghavan 1990

| **Methods** | Study design: Study design: Double-blind Cluster randomized trial conducted between January 1987, and January 1989, in one of the backward districts of Andhra Pradesh, India.  Study duration:12 months |
| --- | --- |
| **Participants** | Sample:15,775 children in 84 clusters (villages) were randomised to the treatment groups (7691) and control groups (8084)  Eligibility: Children of age 1-5 years were eligible for entry.  Exclusion: Children with corneal involvement were given immediate treatment and dropped from the study; those with mild xerophthalmia were managed in the same way as non-xerophthalmic (normal) children. |
| **Interventions** | Intervention: Children in the treatment areas received vitamin A (200 000 IU, maximum of 2 doses) twice six months.  Comparison: Placebo included Arachis oil-the same oil base used in the vitamin A concentrate. |
| **Outcomes** | Mortality, diarrhoea, acute respiratory infections, measles |
| **Other information** | **Child mortality and morbidity rates:**  U5MR was about 130/1000 live births (1989) as per World Bank data. IMR was around 79 per 1,000 live births in 1988-92. (NFHS data)  **Child nutrition status:**  About 6% of the children had night blindness and Bitot spots at baseline. The prevalence of stunting was 63% and wasting was 20% in the year 1989 as per World Bank data.  **Health service delivery:**  Measles immunization coverage was around 42% (1989). In the study area: no distribution of vitamin A done in this area. |

#### **Risk of bias table**

| **Bias** | **Authors' judgement** | **Support for judgement** |
| --- | --- | --- |
| Random sequence generation (selection bias) | Unclear risk | Quote: “The villages were allocated randomly into two groups-treatment and control.”  **Comment:** No sufficient information to provide judgement. |
| Allocation concealment (selection bias) | Unclear risk | Comment: No sufficient information to provide judgement. |
| Blinding of participants and personnel (performance bias) | Low risk | Quote: “The trial was double-blind: the investigators and medical officers did not know which were the treatment and which were the control areas. They were not aware whether the dose they were distributing was vitamin A or placebo.”  **Comment:** Probably done |
| Blinding of outcome assessment (detection bias) | Low risk | Quote: “The trial was double-blind: the investigators and medical officers did not know which were the treatment and which were the control areas. They were not aware whether the dose they were distributing was vitamin A or placebo. ..The same investigators collected mortality data during the three-monthly home visits by questioning parents about each child.  **Comment:** Probably done |
| Incomplete outcome data (attrition bias) | Unclear risk | **Comment:** Unequal children (397 in treatment and 638 in the placebo group) received no dose. Also, out of total randomised still some children were missing for which no information is provided. |
| Selective reporting (reporting bias) | High risk | **Comment:** incidence of infections outcome not provided concerning vitamin A and control groups but provided information on the association between diseases and xerophthalmia |
| Recruitment bias | Unclear risk | Quote: No information was provided to form a judgement. |
| Baseline imbalance | Unclear risk | Quote: The two groups were similar at baseline concerning income status, distribution of weight for age, and crude birth and death rates. The distribution of children according to age and sex was similar in the two groups.  Comment: Although no difference between the groups has been indicated, no data was provided. |
| Loss of clusters | Unclear risk | Comment: No information was provided to form a judgement |
| Incorrect analysis | Unclear risk | Comment: Insufficient information on analysis to allow for judgement |
| Comparability with RCTs randomizing participants | Low risk | The effect of Vitamin A in cluster RCT is unlikely to be different than the effect in RCT. |
| Other bias | High risk | Unequal children (397 in treatment and 638 in the placebo group) received no dose of Vitamin A and placebo. |

### West 1991

| **Methods** | Study design: Cluster randomized double-masked placebo-controlled community trial |
| --- | --- |
| **Participants** | Eligibility: children aged between 0 and 5 years were eligible for the study.  Sample:28 630 children aged 6-72 months in 261 clusters were recruited. 29 local development units, each containing 9 administrative wards, were selected for the trial. Randomisation was carried out by administrative ward;  Children with xerophthalmia were included. Children who had recently participated in a vitamin A programme were excluded from the study. |
| **Interventions** | Vitamin A supplementation (100,000 IU for 6-11 months and 200,000 IU for children 12 months and older) was administered 1-3 times every 4 months, conducted from September 1989, to December 1990.  Placebo: 300 retinol equivalents (1000 IU) of vitamin A (retinyl palmitate in Arachis oil) |
| **Outcomes** | Mortality, cause-specific mortality, Bitot's spots, night blindness, xerophthalmia |
| **Other information** | **Children mortality and morbidity**  U5MR was 126.2/1000 in the country as per NFHS 1996. IMR was reported to be 82/1000 live births in the trial. The prevalence of diarrhoea according to Nepal Family Health Survey 1996 was 28%.  **Child nutrition status**  The baseline xerophthalmia in the study population was 3%. The prevalence of stunting was 68% and wasting was 6% as per the World Bank data.  **Health service delivery**  Measles immunisation coverage was 57% (1989). In the study area, no Vitamin A supplementation. |

#### **Risk of bias table**

| **Bias** | **Authors' judgement** | **Support for judgement** |
| --- | --- | --- |
| Random sequence generation (selection bias) | Unclear risk | Quote: "After a random start, each of the 261 wards was systematically allocated to one of four coded batches of supplements. Two codes contained a standard 60 000 ug retinol equivalent (200 000 IU) dose of vitamin A and two served as control, containing 300 retinol equivalents (1000 IU) of vitamin A"  **Comment:** the process by which the wards were randomized is not clear. |
| Allocation concealment (selection bias) | Unclear risk | Quote: "Both the investigators and communities were masked to the random assignment."  **Comment:** No further information on whether the allocation to treatment and control group was concealed. Also, it was not clear whether allocation took place before or after treatment group assignment was known. |
| Blinding of participants and personnel (performance bias) | Low risk | Quote: "The supplements were given as single-dose gelatin capsules of identical taste and appearance."  **Comment:** Blinding of participants was done, probably participants were not known about the capsule type |
| Blinding of outcome assessment (detection bias) | Low risk | Quote: "The supplements were given as single-dose gelatin capsules of identical taste and appearance."  **Comment:** Probably done. |
| Incomplete outcome data (attrition bias) | Unclear risk | Quote: "All analyses were carried out on an intention-to-treat basis. Computed mortality rates were based on child-years of observation." Quote: " . . . all children living in wards which received high dose vitamin A every 4 months were considered to have been treated with vitamin A, and all children living in wards which received placebo were considered 'untreated.' "  **Comment:** the rates of withdrawal were balanced between the treatment groups and the data were analysed based on patient-years of observation. The unclear reasons for withdrawals, variable duration of follow-up due to more than recruitment cycle and the low rate of mortality about the withdrawal rates mean that it is uncertain whether the study is at risk of attrition bias. |
| Selective reporting (reporting bias) | Low risk | **Comment:** complete data for all time points were available for review. The last available observation reported in a follow-up article gave a RR for mortality slightly higher than that for the 12-month data given in the primary study report (0.74 versus 0.7). |
| Recruitment bias | Low risk | Quote: "First, each village was visited to identify households containing children aged 0-5 years and to mark their dwellings. Within 2 days the village was visited by the full team. Enumerators visited every house containing preschool children, collected socioeconomic, demographic, and medical data, and rounded up children at a central point for their clinical examination." |
| Baseline imbalance | Low risk | **Comment:** Tables I and II show a similar % of baseline characteristics in control and intervention |
| Loss of clusters | Unclear risk | **Comment:** No information about the loss of clusters |
| Incorrect analysis | Low risk | Quote: "Computed mortality rates were based on child-years of observation. Relative risks were derived with all variance and 95% confidence interval estimates adjusted to account for the design effective, the ward rather than the individual serving as the unit of treatment allocation. Mortality rates and relative risks were calculated across strata of age, sex, and nutritional status. A Poisson regression mode119 was fitted to estimate the effect of vitamin A on mortality with simultaneous adjustment for each of these factors."  **Comment:** Cluster adjustment probably done. |
| Comparability with RCTs randomizing participants | Low risk | The effect of Vitamin A in cluster RCT is unlikely to be different than the effect in RCT. |
| Other bias | Unclear risk | No sufficient information to permit judgement. |
