# Supplementary material for: Is routine Vitamin A supplementation still justified for children in Nepal? Trial synthesis findings applied to Nepal national mortality estimates
Source: PLoS One. 2022 May 18;17(5):e0268507. doi: 10.1371/journal.pone.0268507 (PMC9116662; doi:10.1371/journal.pone.0268507)
Supplement: Attachment — Submitted filename: Response to Reviewers-Final to Submit.docx [file pone.0268507.s011.docx]

Dear reviewers,

Thank you for your time to review our manuscript. We also appreciate your comments and input to improve the manuscript for further. We have revised the manuscript according the comments received. Please see the below table as the summary of the revision and responses to the comments that you have provided.

| **Comments** | **Response** | **Revised contents** |
| --- | --- | --- |
| 1. The title states that “evidence analysis using contemporary estimates”. The authors attempted to update the earlier meta-analysis with more recent studies, however, there was only one study which was not included in the earlier meta analysis, Eventually this one additional study was also dropped from the meta-analysis because it did not fulfil the criteria of inclusion. Therefore although the authors claim review of recent studies, eventually there are no more recent studies included in the Cochrane review and the title may not be justified. | We agree with the reviewer that, although we attempted to update the meta-analysis with recent studies, no recent studies were available so as suggested the title of the review has been revised.  We revised the title as:  “Is routine Vitamin A supplementation still justified for children in Nepal? Trial synthesis findings applied to Nepal national mortality estimates” | Page# 1 Lines#1-2 |
| 2. This study chose only a subset of the studies in cluded in the Cochrane review. The criteria for selection of studies for re-analysis is not clear and it seems to have not considered a list of studies without clear justification. Eg: Donnen 1998 estimate of all cause mortality is presented in the Cochrane review although this study is dropped from the current analysis. The paper by Donnen et al did report 5.4% mortality in the Vit A group compared to 9% in control group resulting in a risk ratio of 0.6 which is reported in the Cochrane review. This was not a primary outcome of the study, but this is not sufficient justification for dropping the study. Hence the exclusion of studies should be re-considered. | We have considered only those studies that are RCTs and have explicitly reported mortality outcome data and have excluded the studies accordingly. However, as suggested by the reviewer, we have re-considered those studies which we had excluded. And upon re-consideration, we have now considered Donnen 1998 in the included studies. However, upon re-consideration other studies (Andersen 2018, Barreto 1994, Dibley 1996, Lin 2008, Chowdhury, 2002) remain as excluded. | Page# 8  Lines#140-143 |
| 3. The authors state that no study has a baseline U5mortality estimate close to that of Nepal which is 28 in 1000. But the next line states that “Thus, the mortality estimate most similar to current values in Nepal gives an estimate of 4% (RR 0.96, 95% CI 0.90 to 1.03) reduction in child mortality with VAS.” Which study is represented here? | We categorized baseline U5MR in four categories for sub-group analysis   - U5MR (30-60): no studies - U5MR (60-90): no studies - U5MR (90-120): RR 0.96, 95% CI 0.90 to 1.03 - U5MR>120: RR 0.75 [0.68, 0.83]   No studies included had a baseline U5MR that represented the current U5MR (28/1000) in Nepal as there were no studies in the sub-categories (U5MR 30-60 and U5MR 60-90). Almost all included studies had a baseline U5MR of greater than 90, the sub-group (90-120) gave an effect estimate of (RR 0.96, 95% CI 0.90 to 1.03). So, the estimate represented here is from the sub-group analysis of U5MR (baseline) from the strata U5MR (90-120) which included studies that had baseline U5MR between 90-120. This sentence has been revised to make it clear. | Page# 15  Lines#268-274 |
| 4. It might be useful to do a separate analysis of the Nepali studies considered in the Cochrane review as has been done in the recently published Indian Pediatrics paper (<https://www.indianpediatrics.net/epub092021/RP-00372.pdf>) | This is possible, but we consider this may confuse: a) evidence to decision frameworks depend on meta-analysis, rather than the findings of country trials; and b) quality of the evidence obtained from Nepal based studies is limited due to the risk of bias (concerns related to allocation concealment), and thus primarily considered the best estimate of effect obtained from high quality adequately concealed studies included in the Cochrane review for estimating the potential impact of supplementation in Nepal. We have noted in the discussion that the Nepal trial data are consistent with the overall analysis. We added our argument in the discussion as “Thus the conclusion of the Cochrane review that “Vitamin A supplementation is associated with a clinically meaningful reduction in morbidity and mortality in children” is probably not correct: we recommend national policy makers use the estimates from the review applied to their national data to determine themselves the potential public health benefits of continuing routine supplementation”. | Page# 18  Lines#319-323 |
